# Supplementary material for: Benchmarking long-read variant calling in diploid and polyploid genomes: insights from human and plants
Source: BMC Genomics. 2026 Jan 15;27:46. doi: 10.1186/s12864-025-12259-5 (PMC12809965; doi:10.1186/s12864-025-12259-5)
Supplement: Supplementary file 1 — Supplementary Material 1. [file 12864_2025_12259_MOESM1_ESM.docx]

**Supplementary Figures**


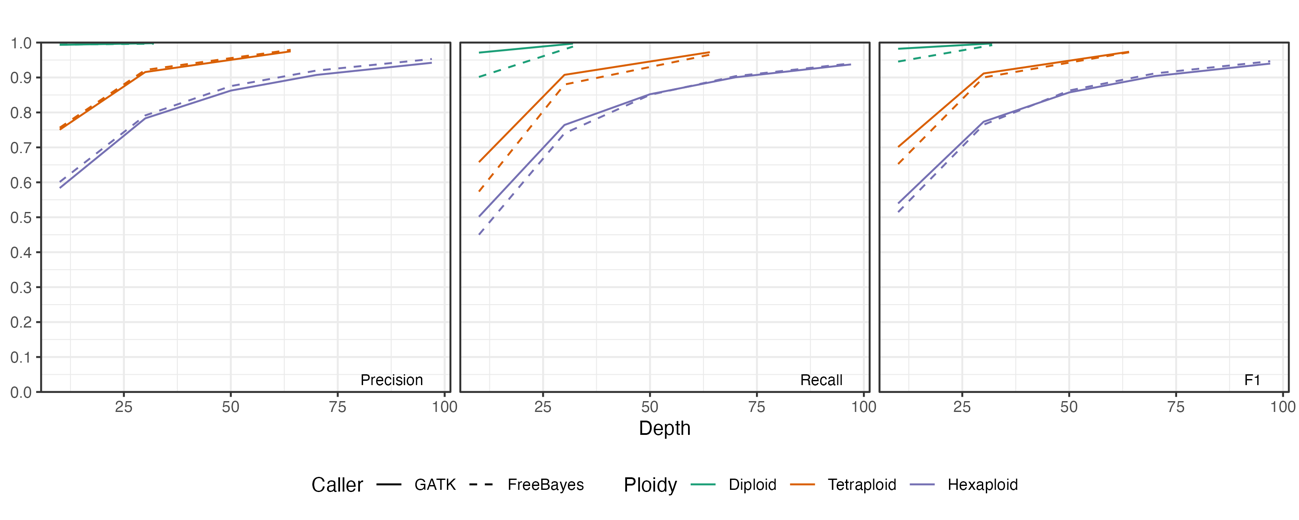


Figure S1 Performance of SNV genotyping on the synthetic human polyploid genome using high-accuracy long reads. Precision, recall, and F1 scores are presented.


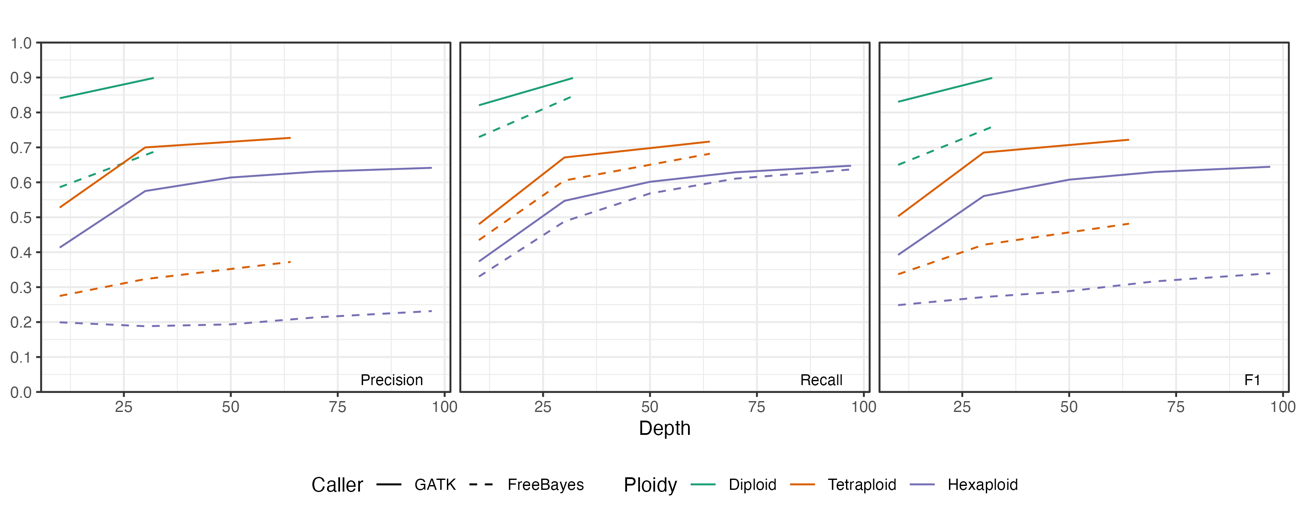


Figure S2 Performance of indel genotyping on the synthetic human polyploid genome using high-accuracy long reads. Precision, recall, and F1 scores are presented.


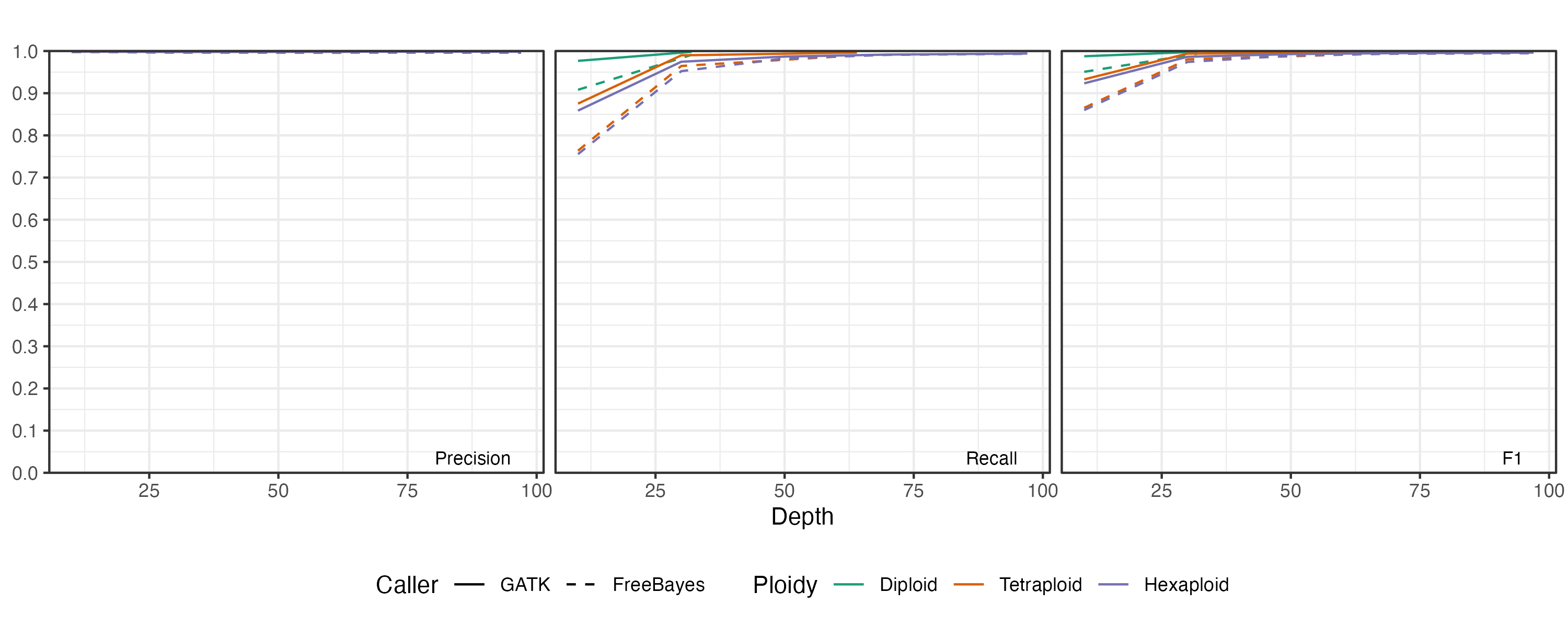


Figure S3 Performance of SNV detection on the synthetic human polyploid genome using high-accuracy long reads. Precision, recall, and F1 scores are presented.


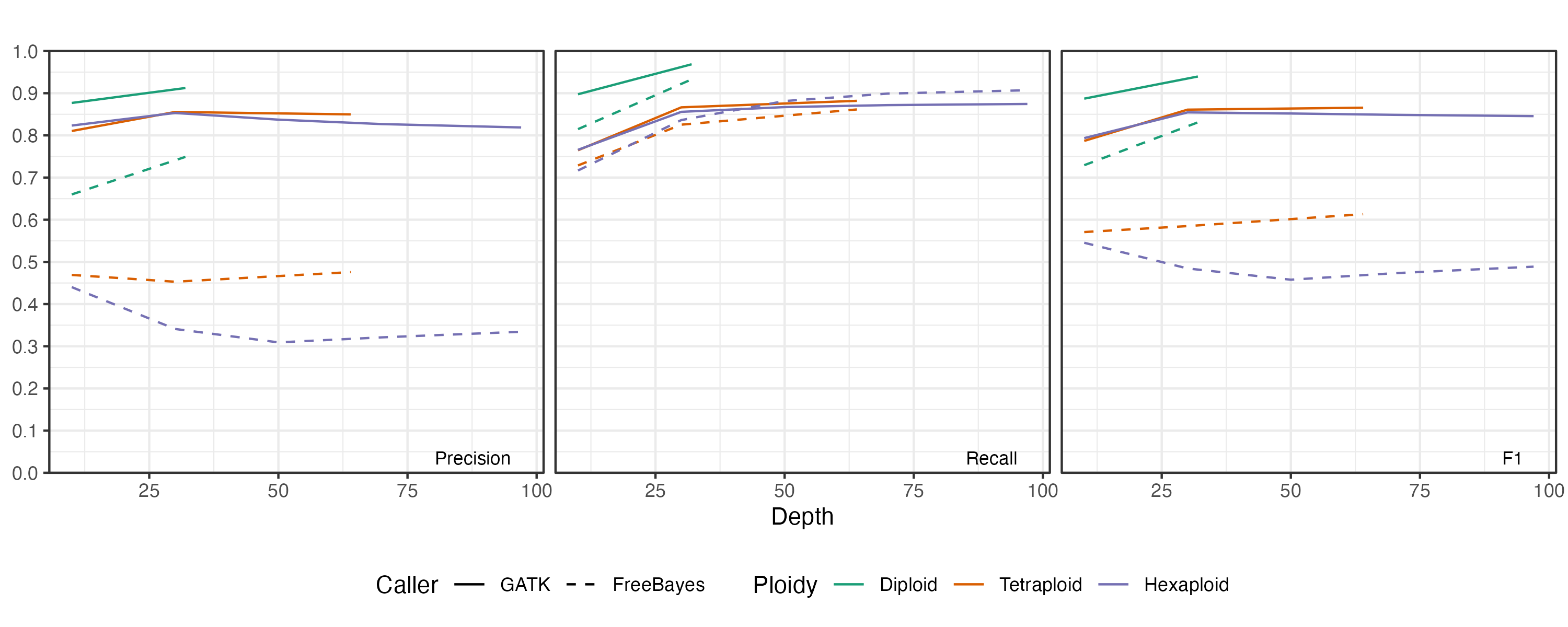


Figure S4 Performance of indel detection on the synthetic human polyploid genome using high-accuracy long reads. Precision, recall, and F1 scores are presented.


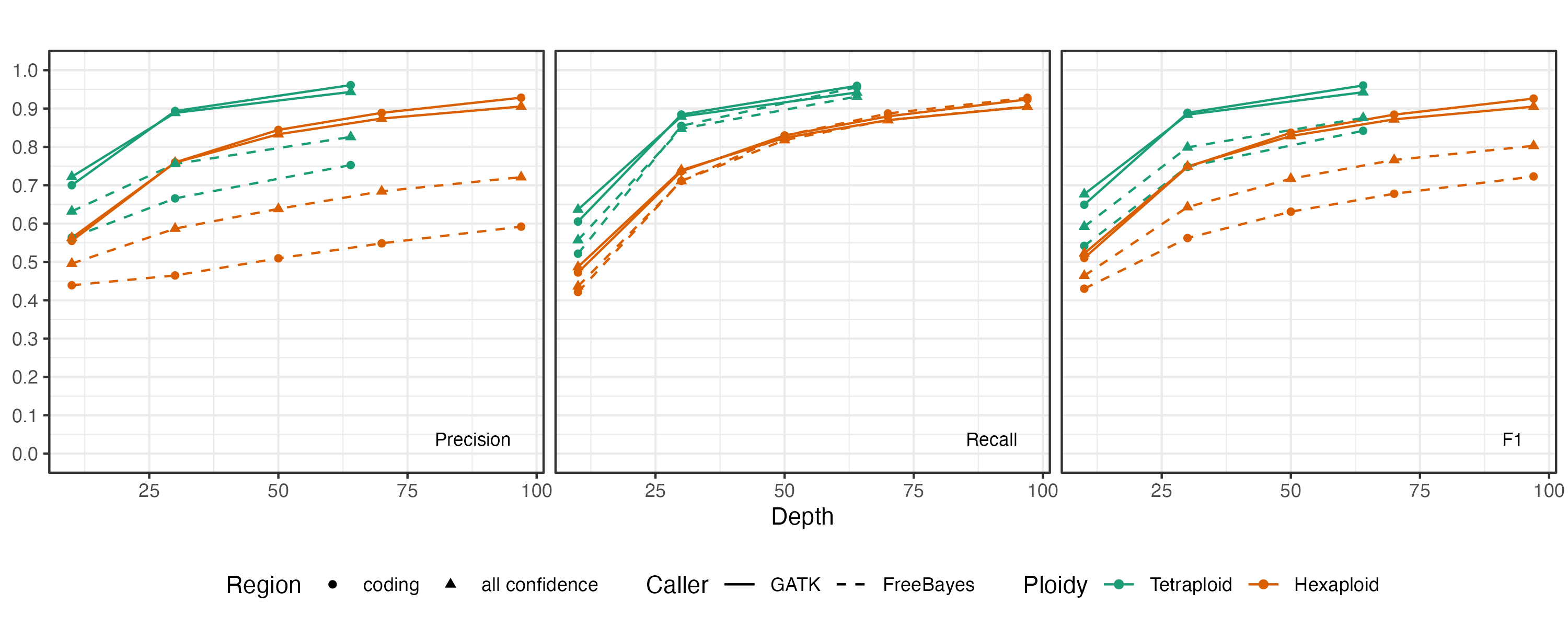


Figure S5 Performance of small variant detection in coding and non-coding regions on the synthetic human polyploid genome using high-accuracy long reads. Precision, recall, and F1 scores are presented.


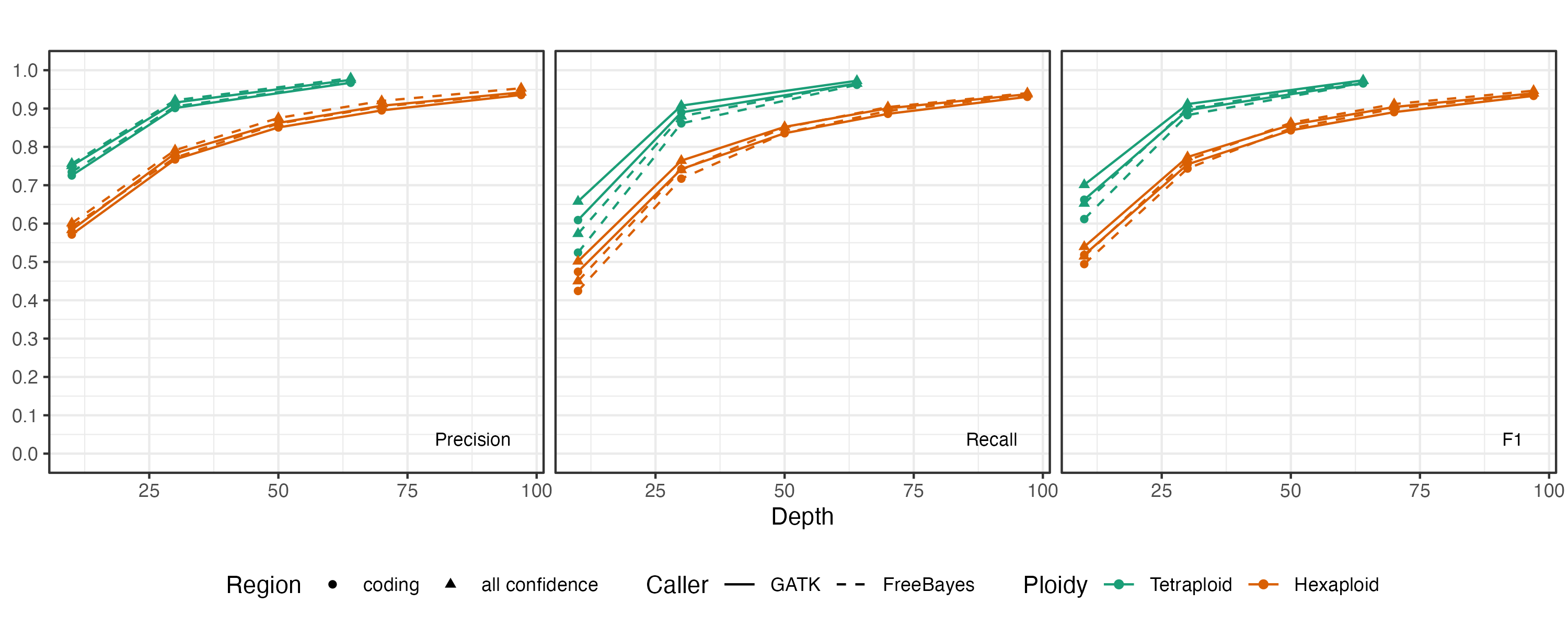


Figure S6 Performance of SNV detection in coding and non-coding regions on the synthetic human polyploid genome using high-accuracy long reads. Precision, recall, and F1 scores are presented.


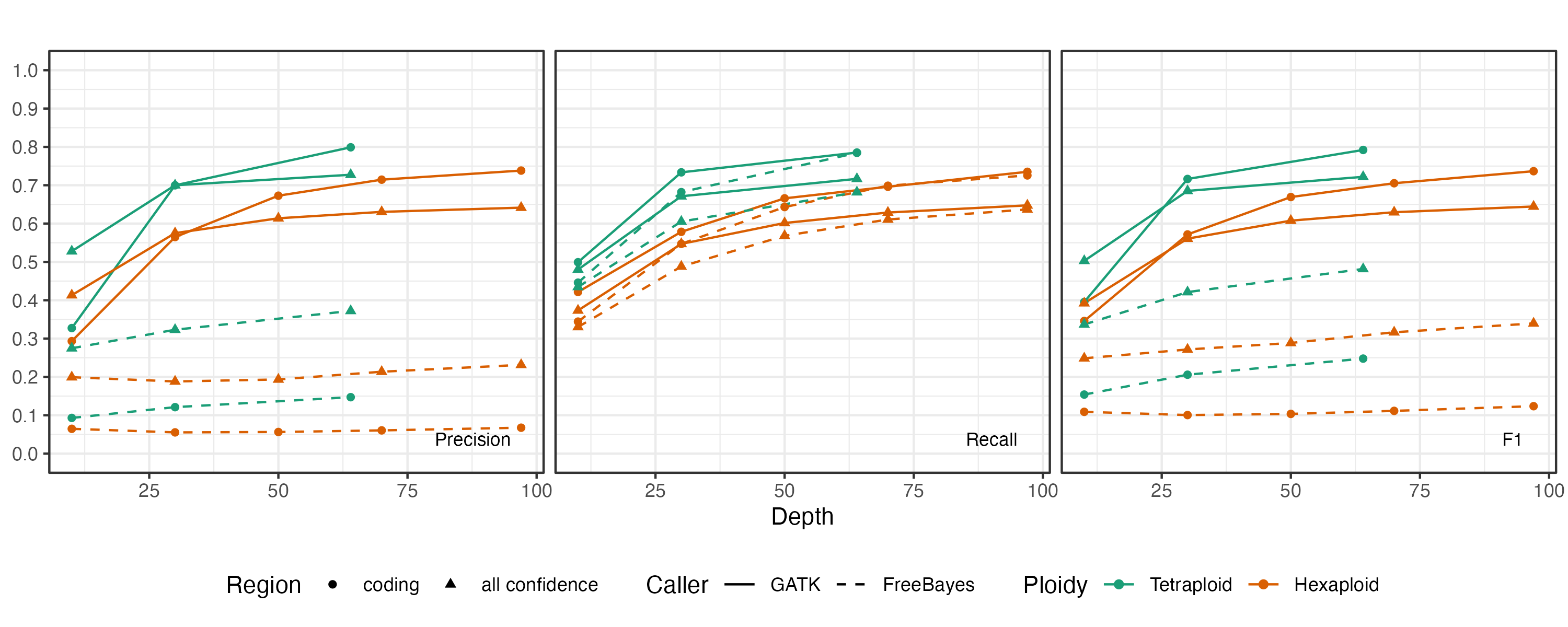


Figure S7 Performance of Indel detection in coding and non-coding regions on the synthetic human polyploid genome using high-accuracy long reads. Precision, recall, and F1 scores are presented.


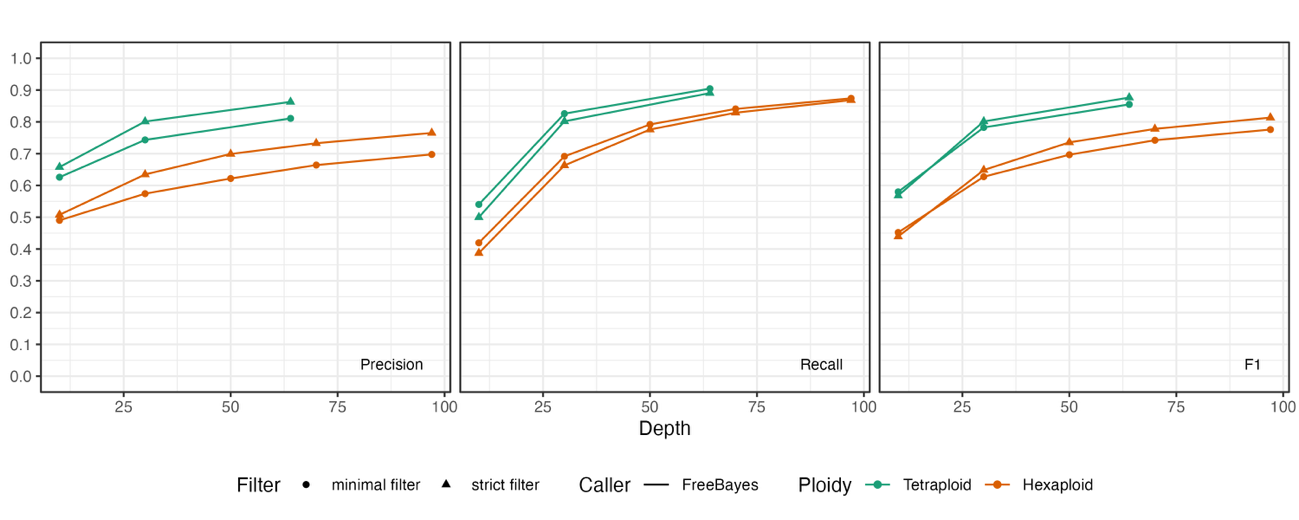


Figure S8 Performance of small variant genotyping by FreeBayes on chromosome 20 of the synthetic human polyploid genome using high-accuracy long reads under minimal and strict filtering. Precision, recall, and F1 scores are presented.


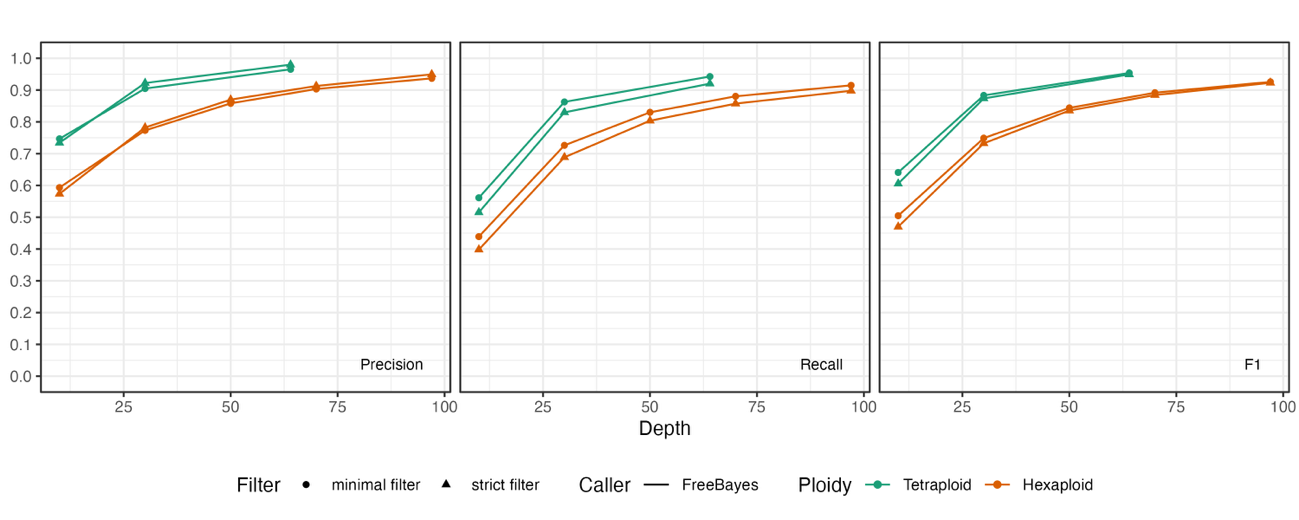


Figure S9 Performance of SNV genotyping by FreeBayes on chromosome 20 of the synthetic human polyploid genome using high-accuracy long reads under minimal and strict filtering. Precision, recall, and F1 scores are presented.


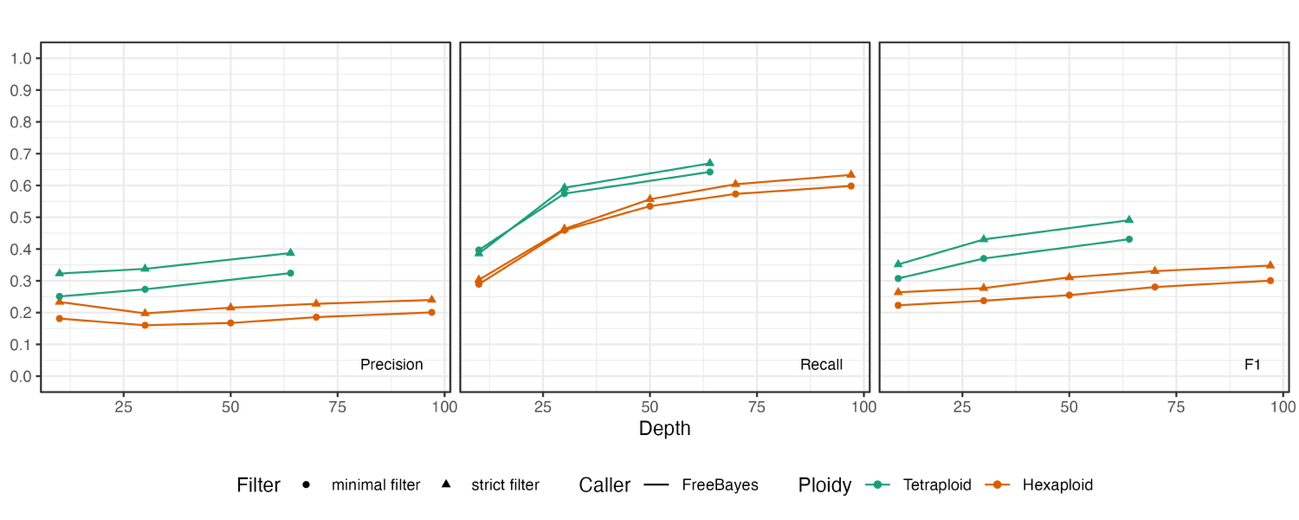


Figure S10 Performance of small variant genotyping by FreeBayes on chromosome 20 of the synthetic human polyploid genome using high-accuracy long reads under minimal and strict filtering. Precision, recall, and F1 scores are presented.


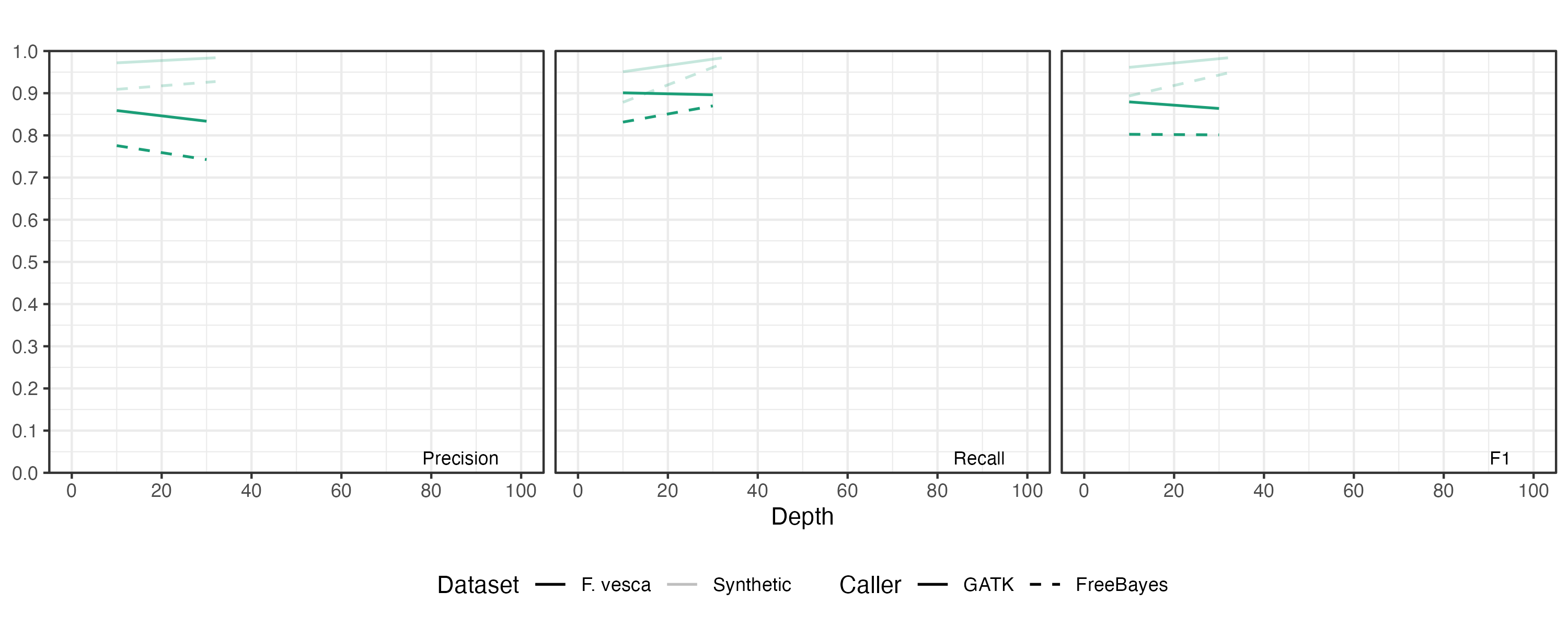


Figure S11 Performance of small variant genotyping on *F. vesca* genome using high-accuracy long reads. Precision, recall, and F1 scores are presented.


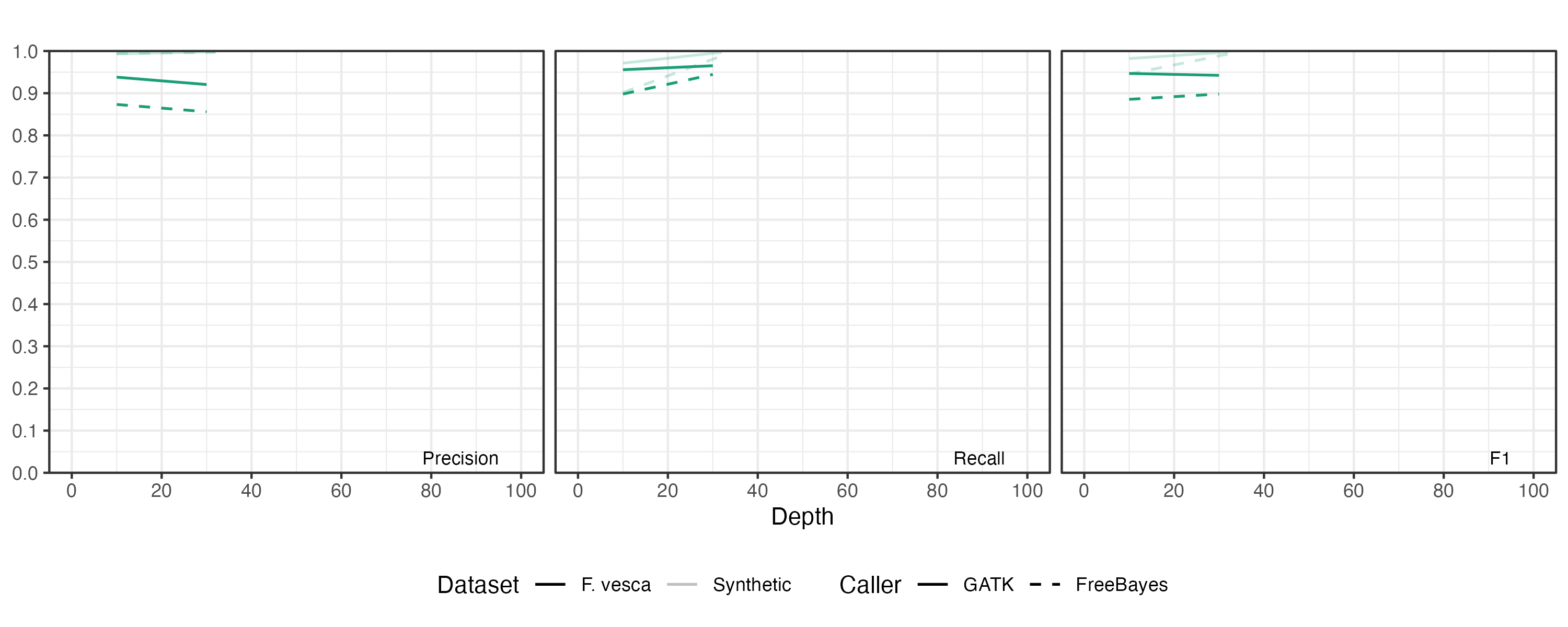


Figure S12 Performance of SNV genotyping on *F. vesca* genome using high-accuracy long reads. Precision, recall, and F1 scores are presented.


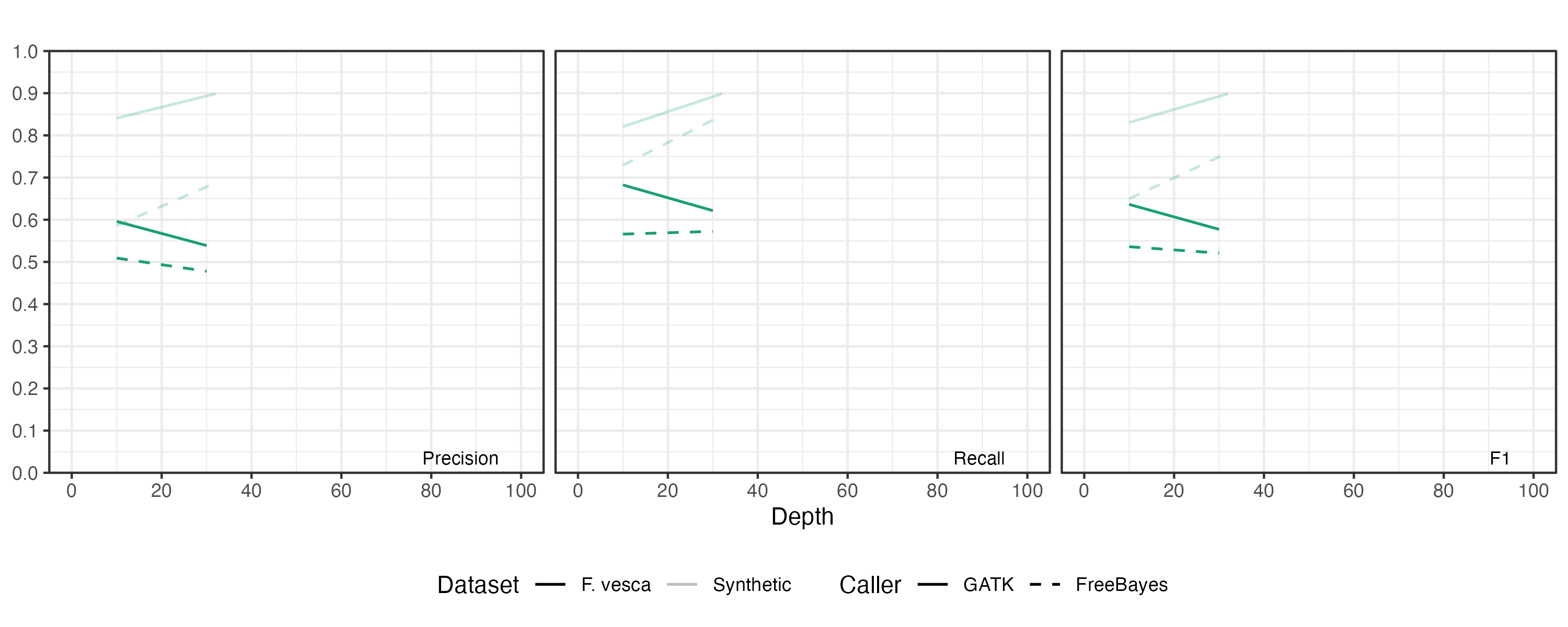


Figure S13 Performance of indel genotyping on *F. vesca* genome using high-accuracy long reads. Precision, recall, and F1 scores are presented.


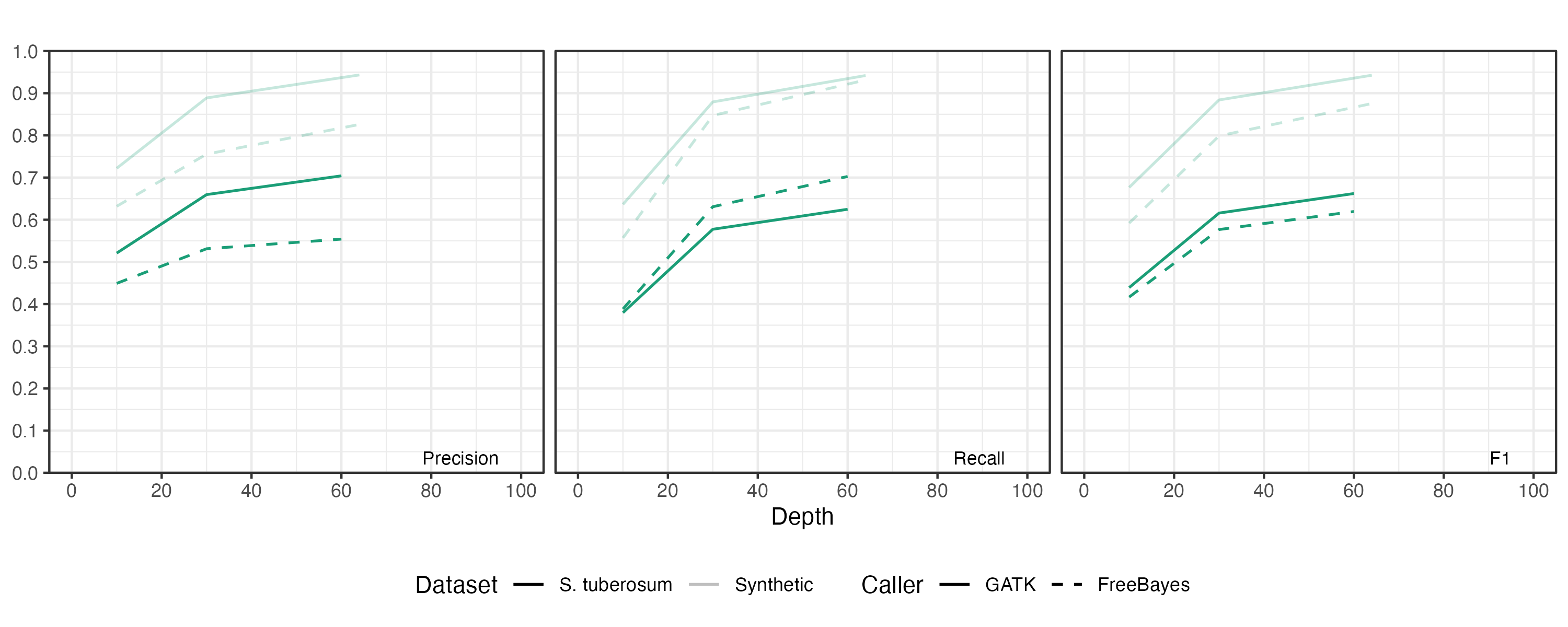


Figure S14 Performance of small variant genotyping on *S. tuberosum* genome using high-accuracy long reads. Precision, recall, and F1 scores are presented.


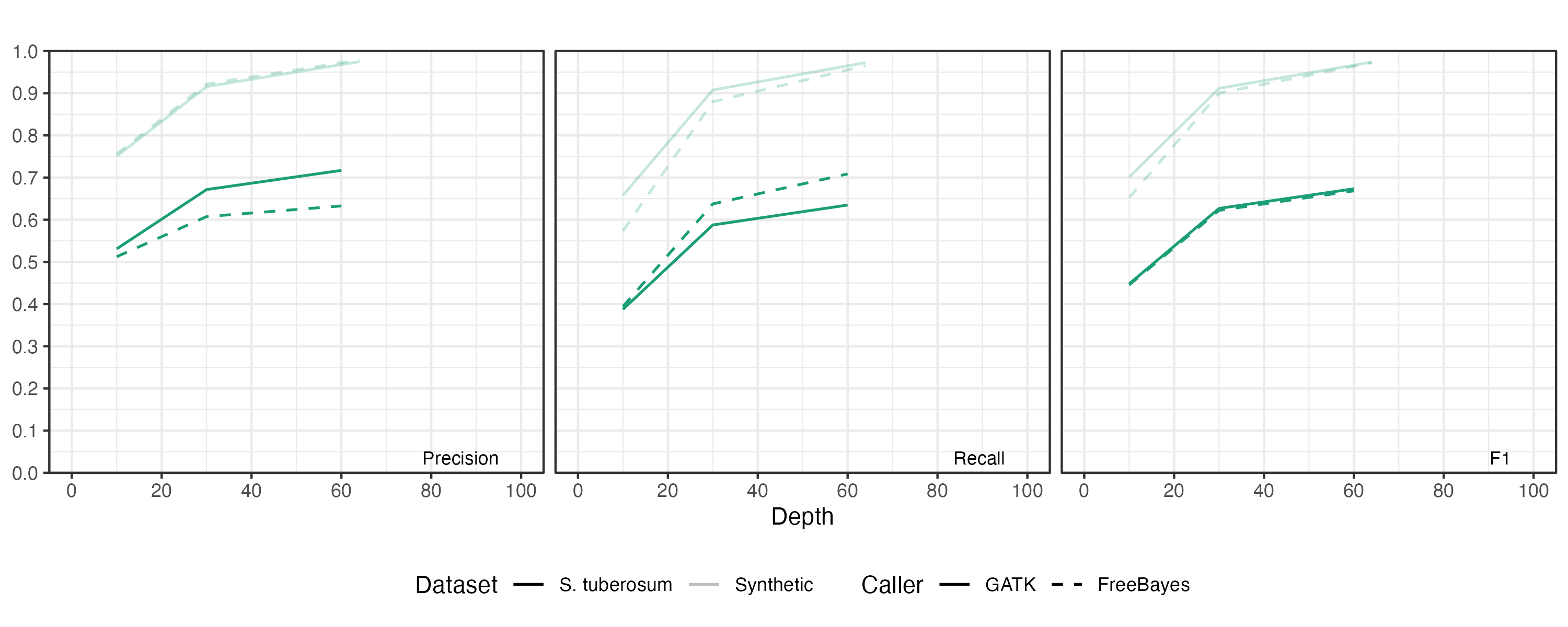


Figure S15 Performance of SNV genotyping on *S. tuberosum* genome using high-accuracy long reads. Precision, recall, and F1 scores are presented.


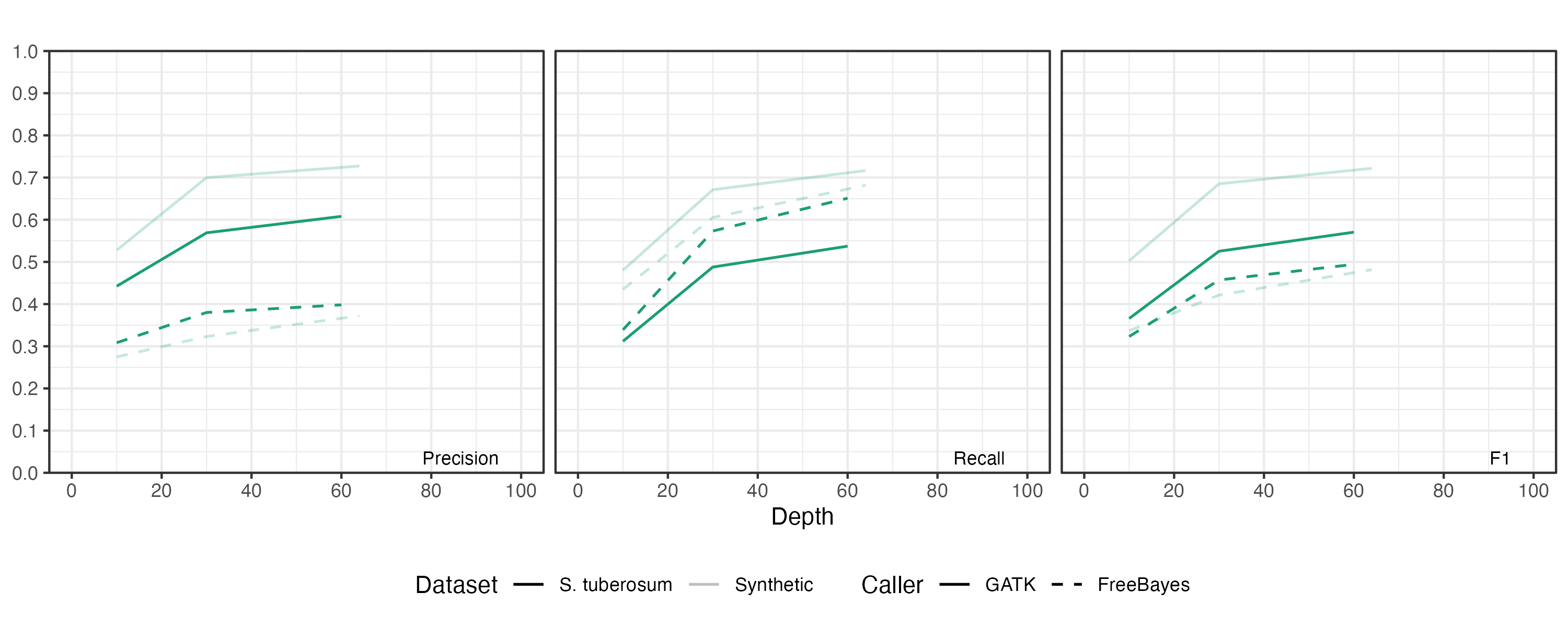


Figure S16 Performance of indel genotyping on *S. tuberosum* genome using high-accuracy long reads. Precision, recall, and F1 scores are presented.


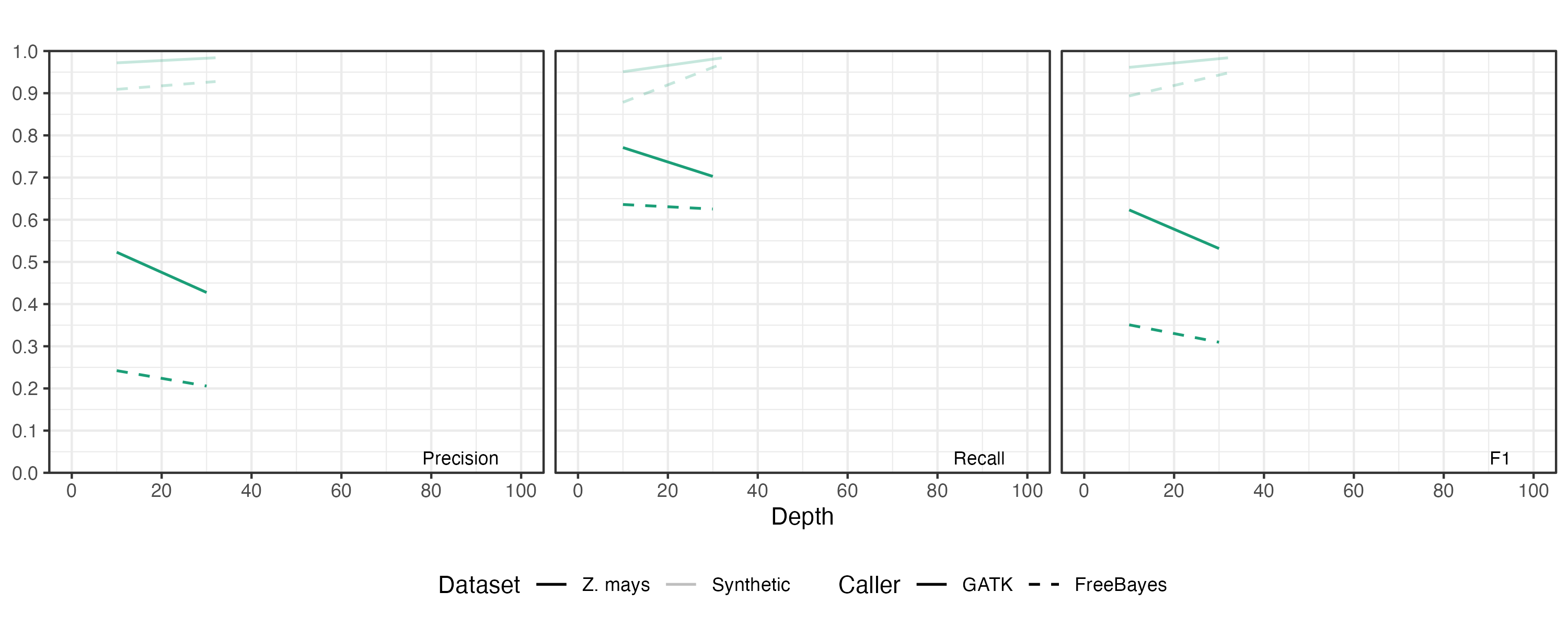


Figure S17 Performance of small variant genotyping on *Z. mays* genome using high-accuracy long reads. Precision, recall, and F1 scores are presented.
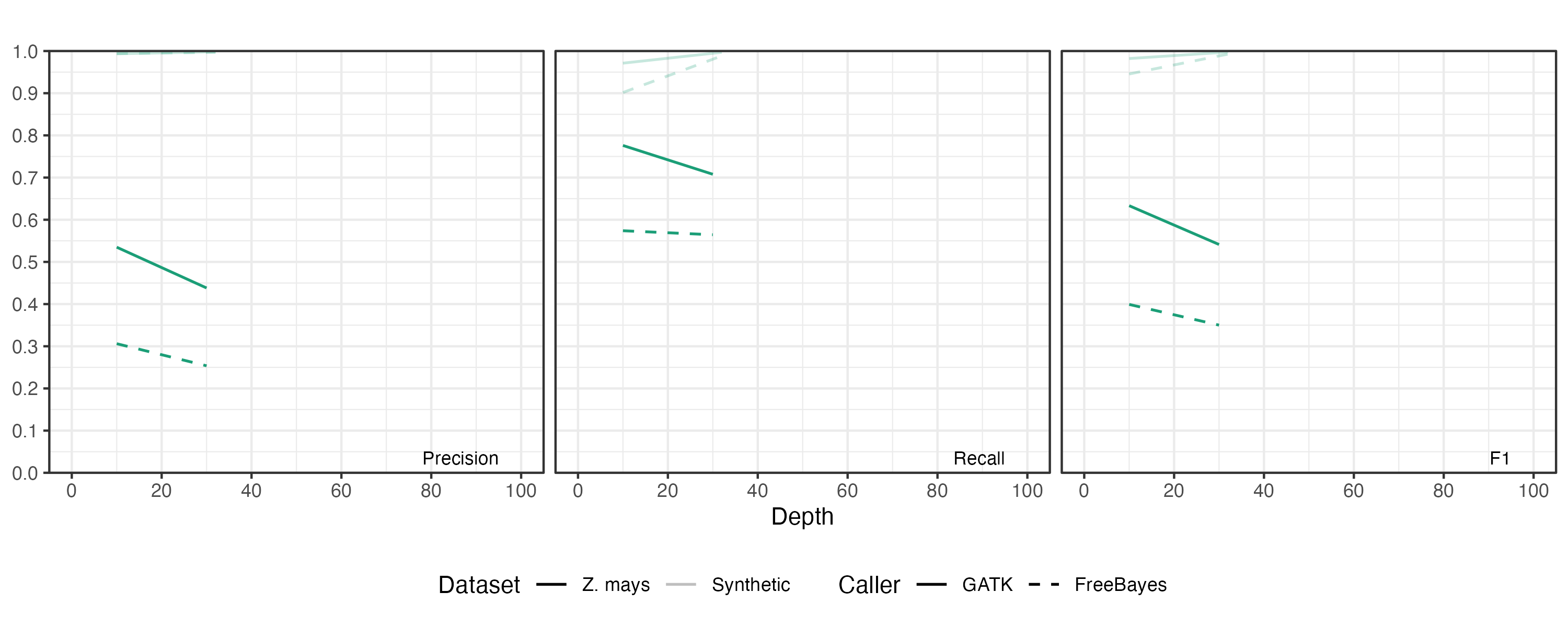


Figure S18 Performance of SNV genotyping on *Z. mays* genome using high-accuracy long reads. Precision, recall, and F1 scores are presented.


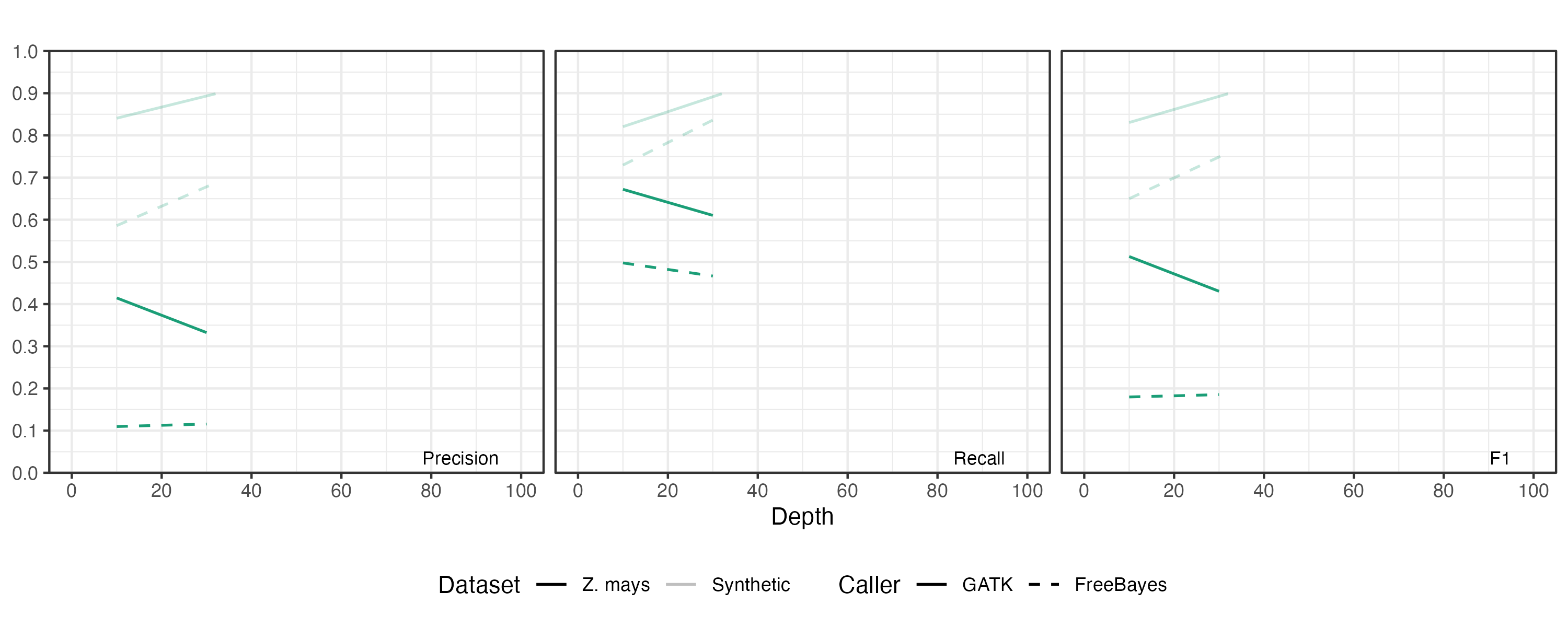


Figure S19 Performance of Indel genotyping on *Z. mays* genome using high-accuracy long reads. Precision, recall, and F1 scores are presented.


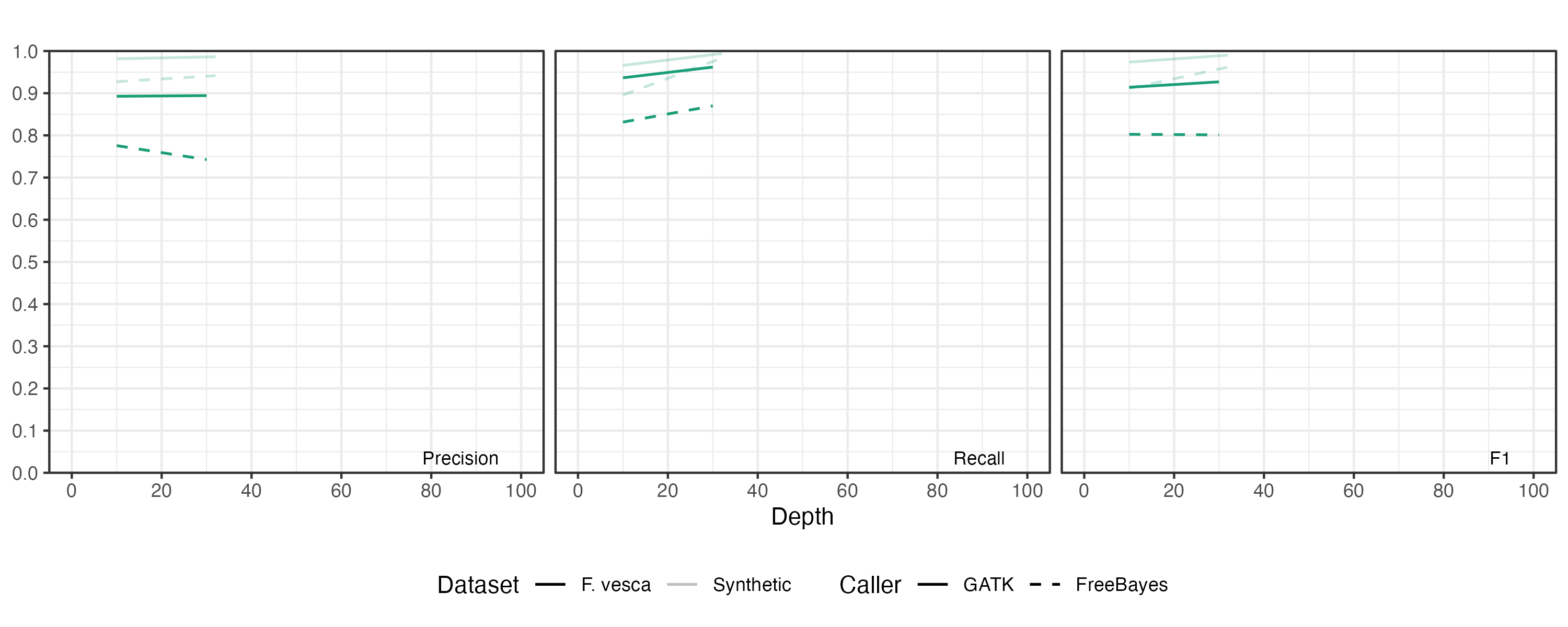


Figure S20 Performance of small variant detection on the *F. vesca* genome using high-accuracy long reads.


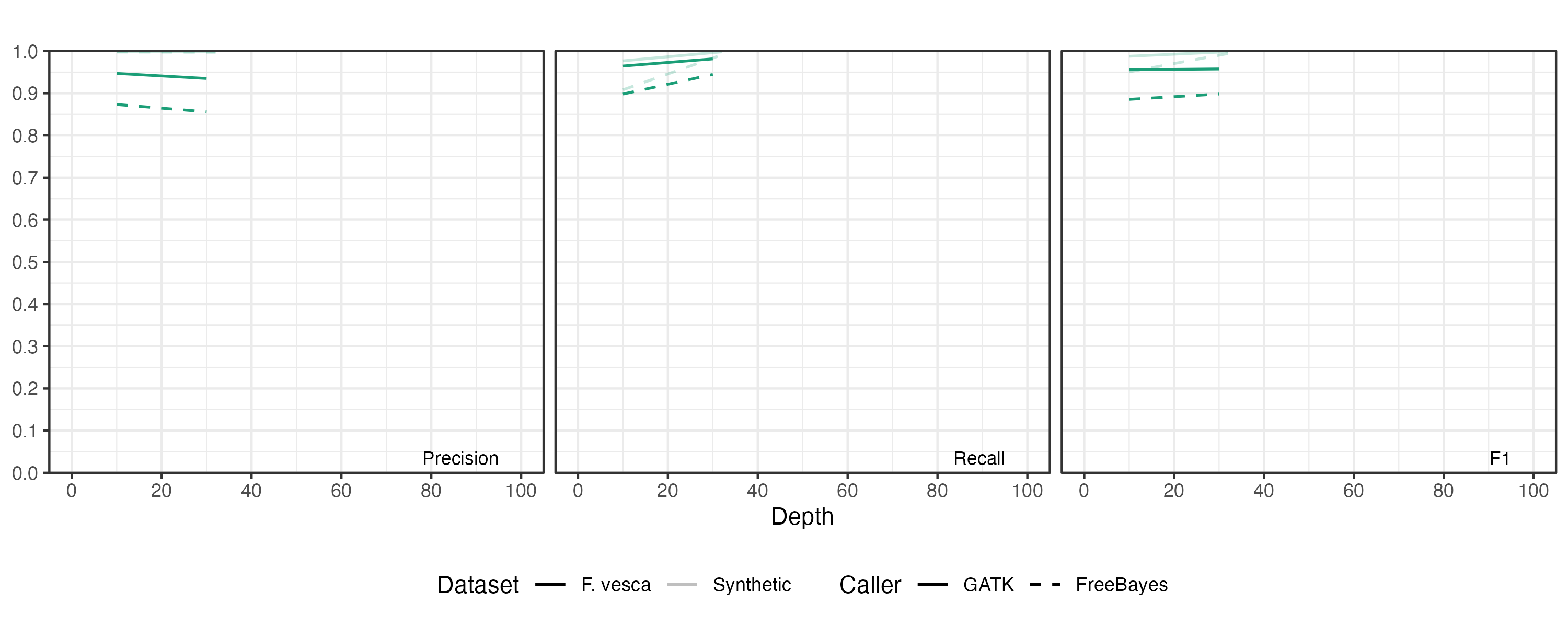


Figure S21 Performance of SNV detection on the *F. vesca* genome using high-accuracy long reads.


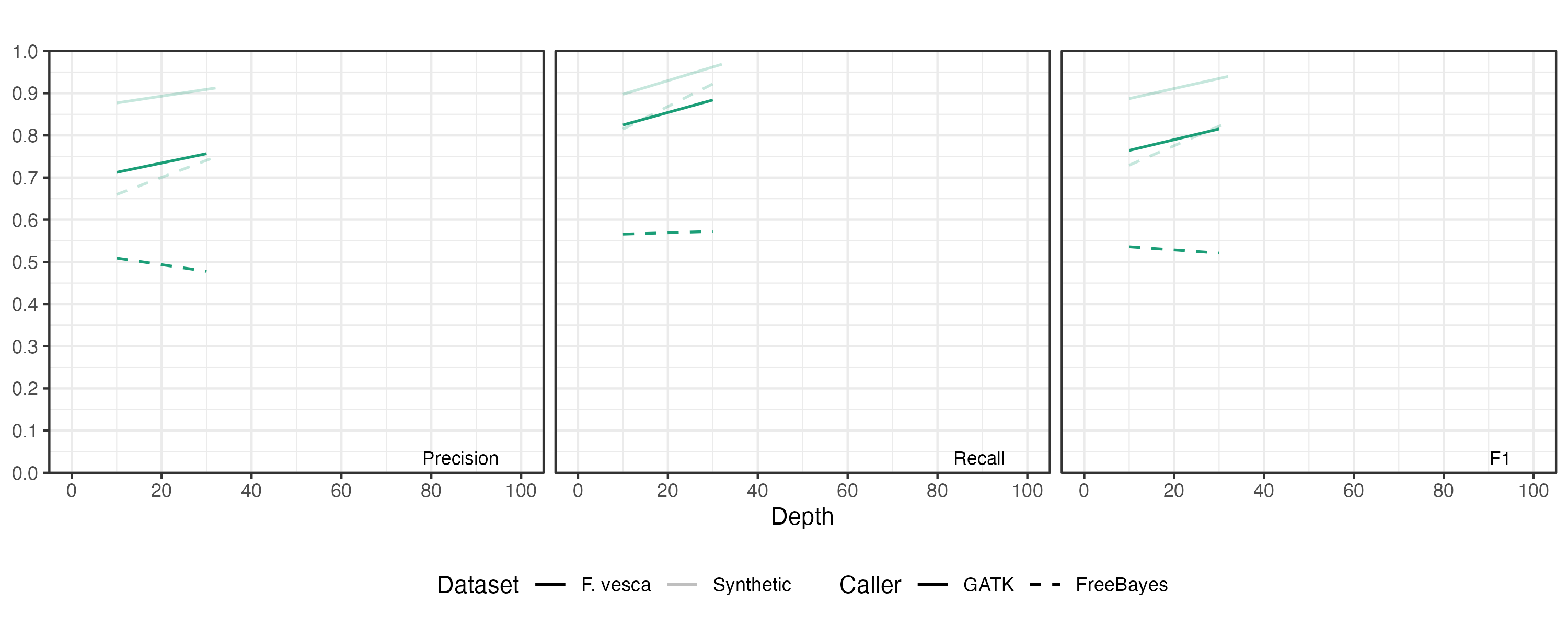


Figure S22 Performance of Indel detection on the *F. vesca* genome using high-accuracy long reads.


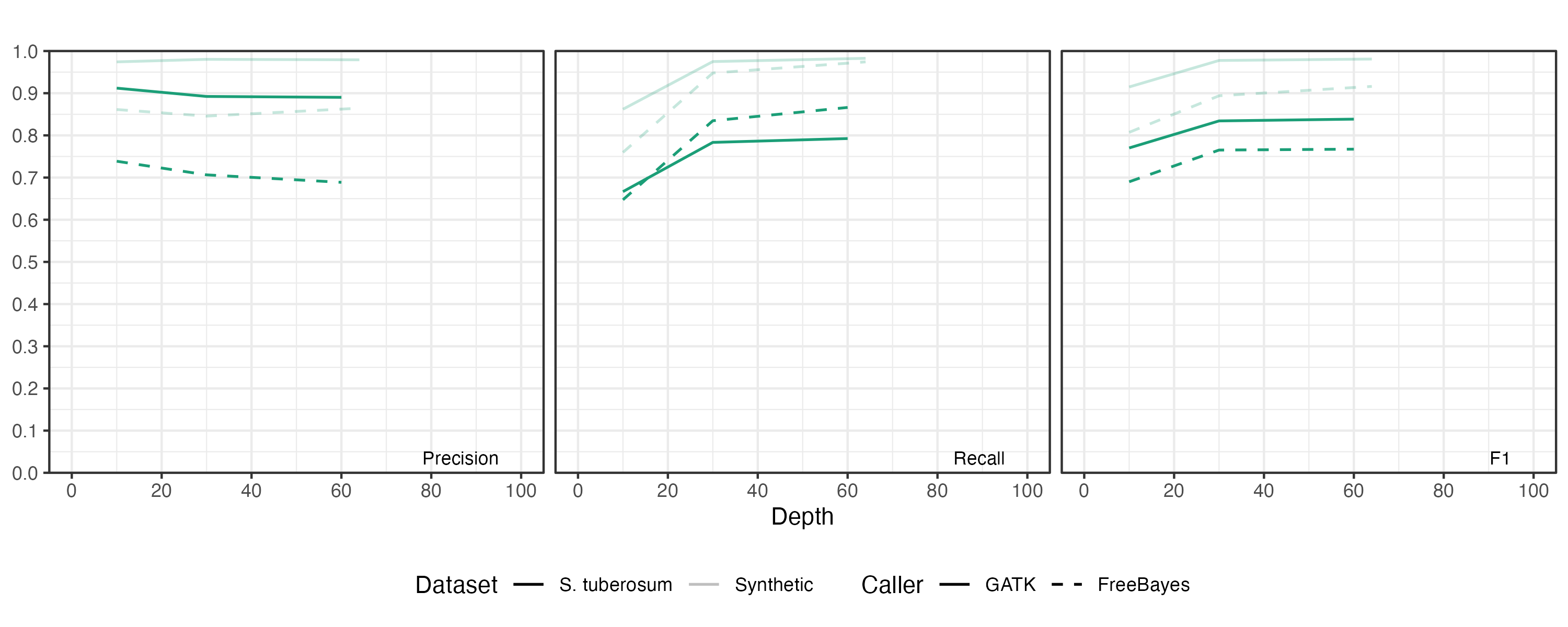


Figure S23 Performance of small variant detection on the *S. tuberosum* genome using high-accuracy long reads.
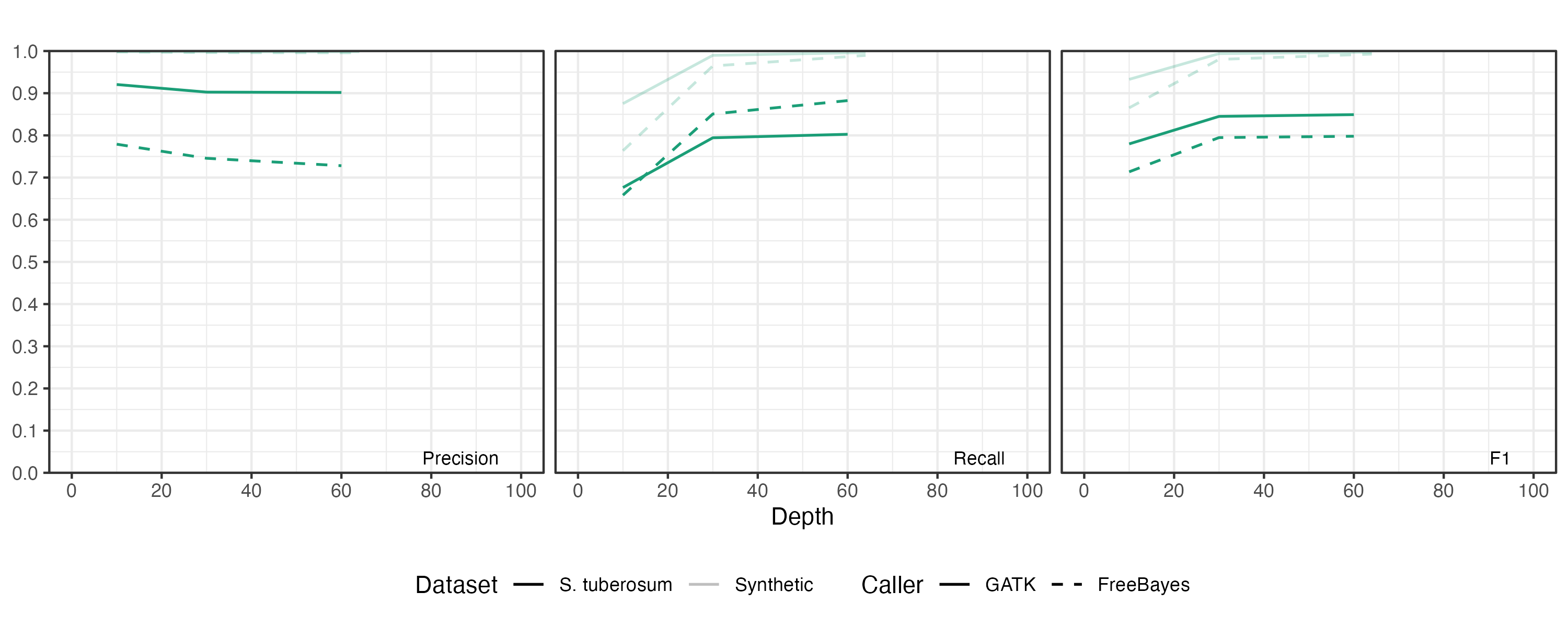


Figure S24 Performance of SNV detection on the *S. tuberosum* genome using high-accuracy long reads.


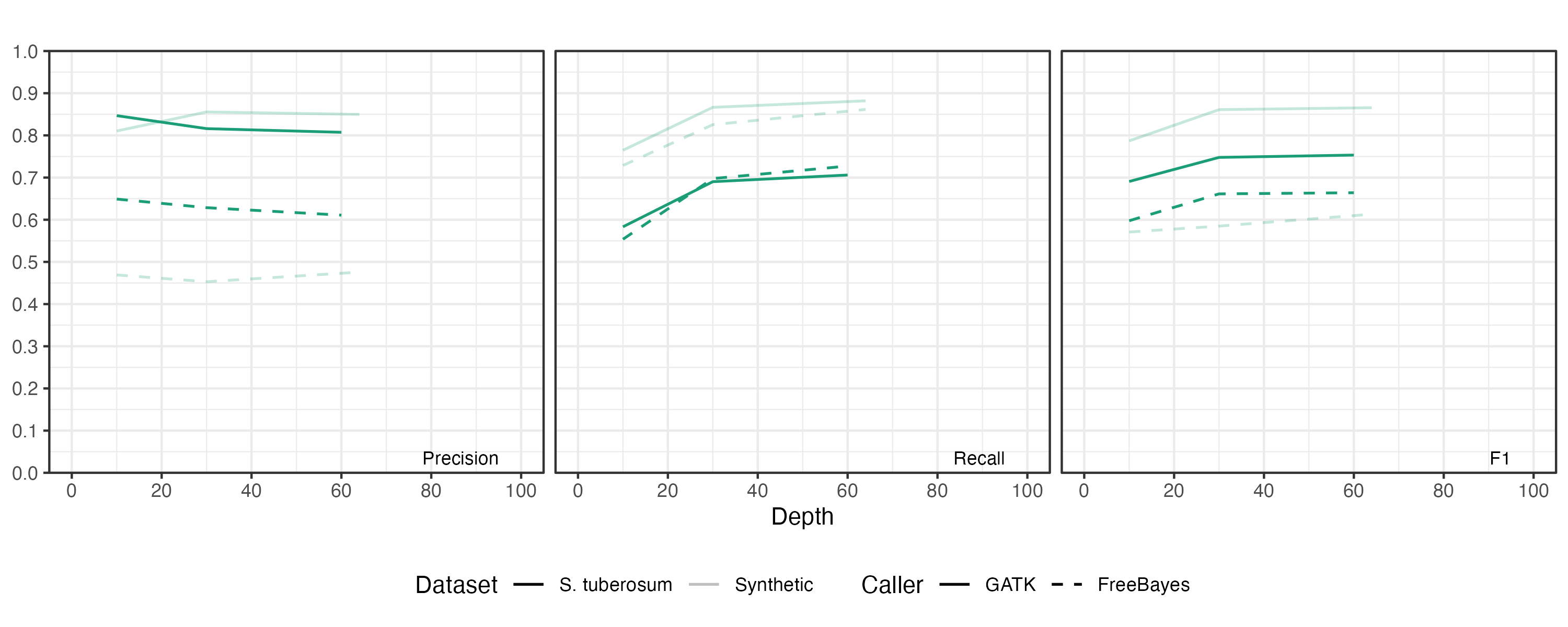


Figure S25 Performance of Indel detection on the *S. tuberosum* genome using high-accuracy long reads.


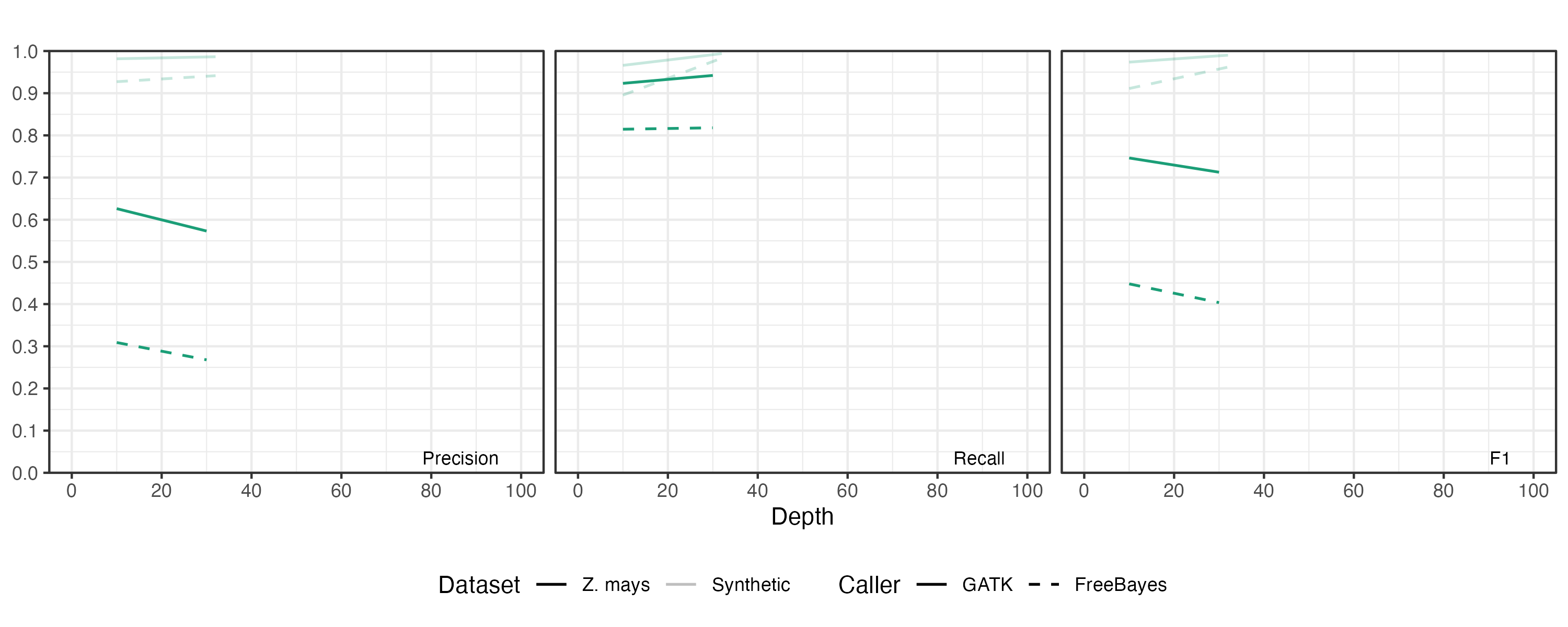


Figure S26 Performance of small variant detection on the *Z. mays* genome using high-accuracy long reads.


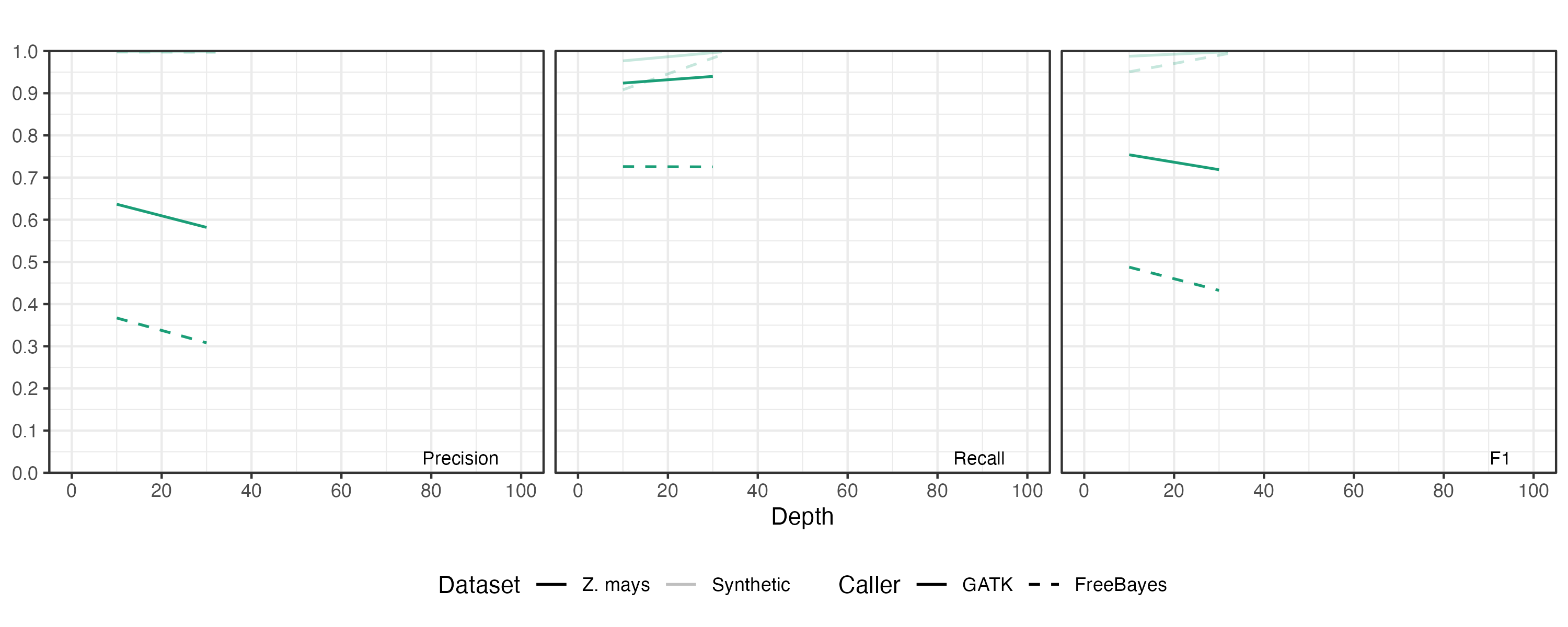


Figure S27 Performance of SNV detection on the *Z. mays* genome using high-accuracy long reads.


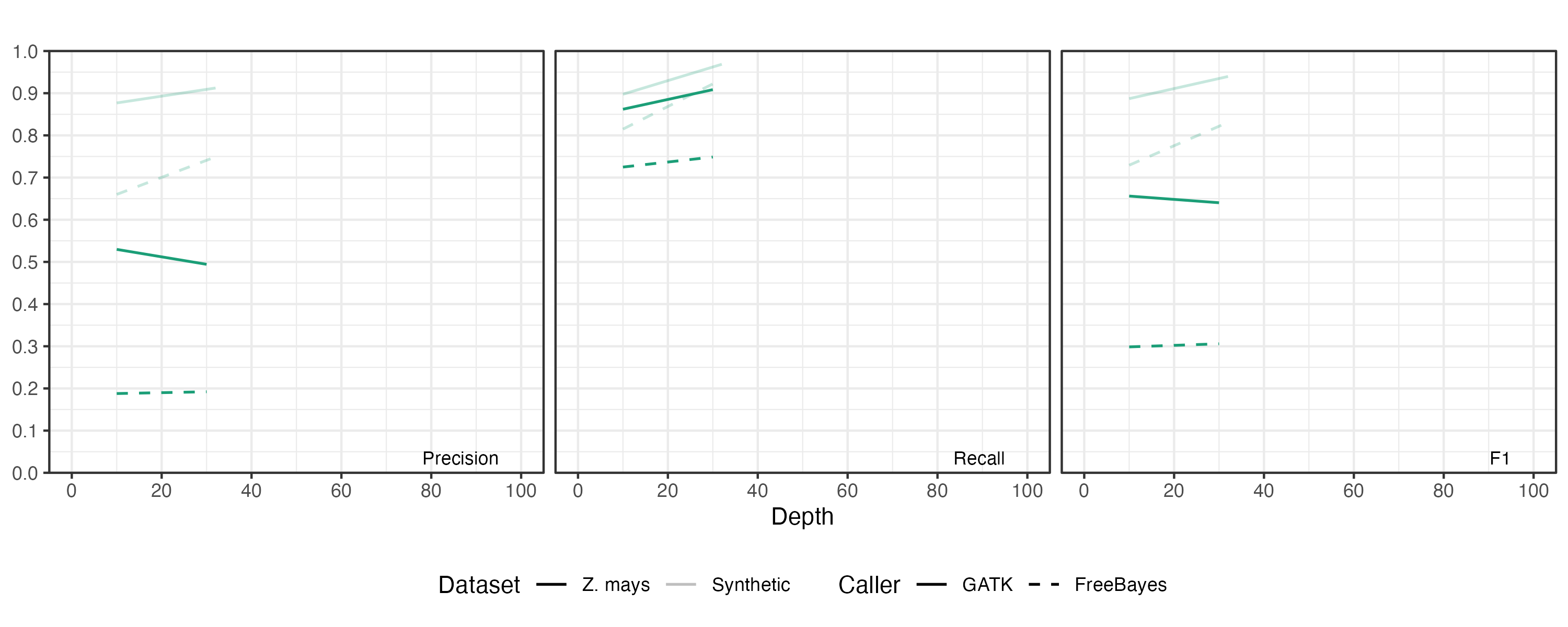


Figure S28 Performance of Indel detection on the *Z. mays* genome using high-accuracy long reads.

Figure S29 Performance of variant genotyping on the human and plant genomes using minimap2 (mm2) and winnowmap2 (wm2). Cross-mapper F1 scores are presented.


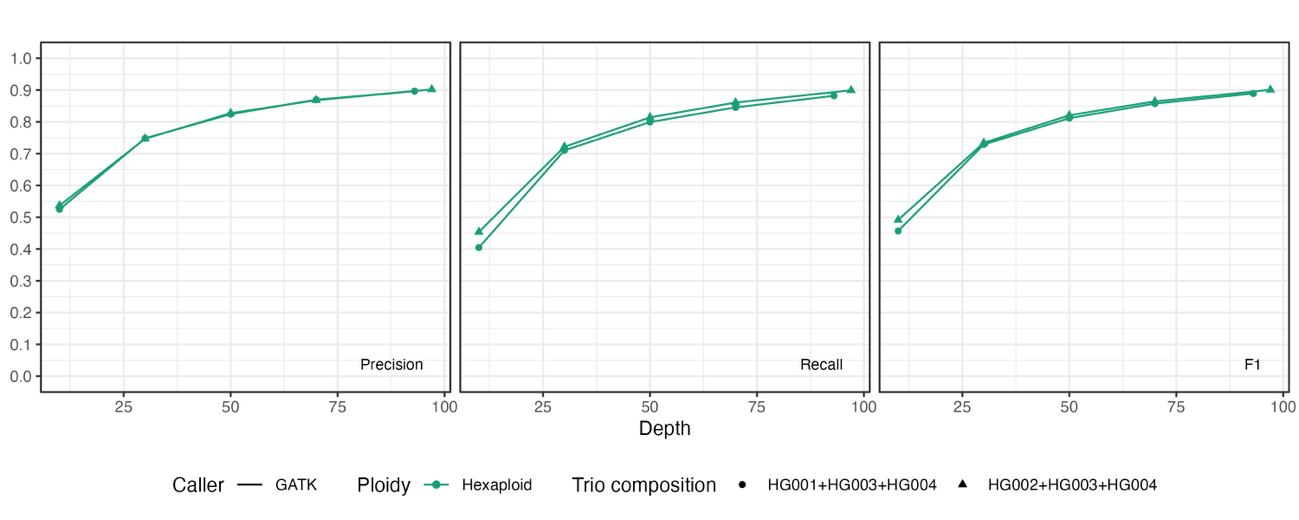


Figure S30 Performance of small-variant genotyping by GATK on chromosome 20 of the synthetic human hexaploid genomes using high-accuracy long reads. Precision, recall, and F1 scores are presented.


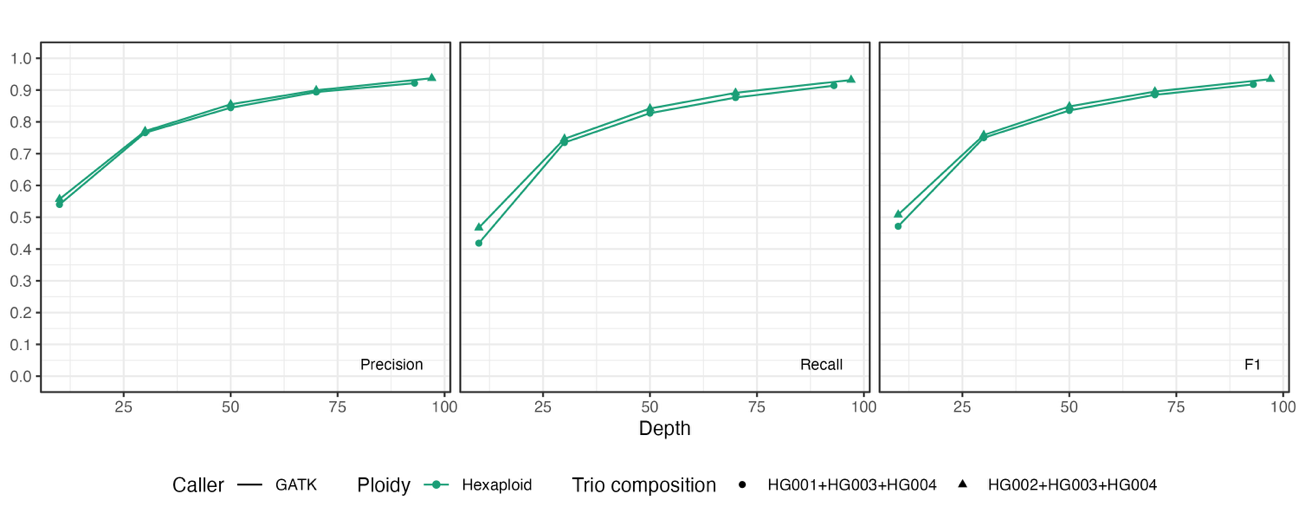


Figure S31 Performance of SNV genotyping by GATK on chromosome 20 of the synthetic human hexaploid genomes using high-accuracy long reads. Precision, recall, and F1 scores are presented.


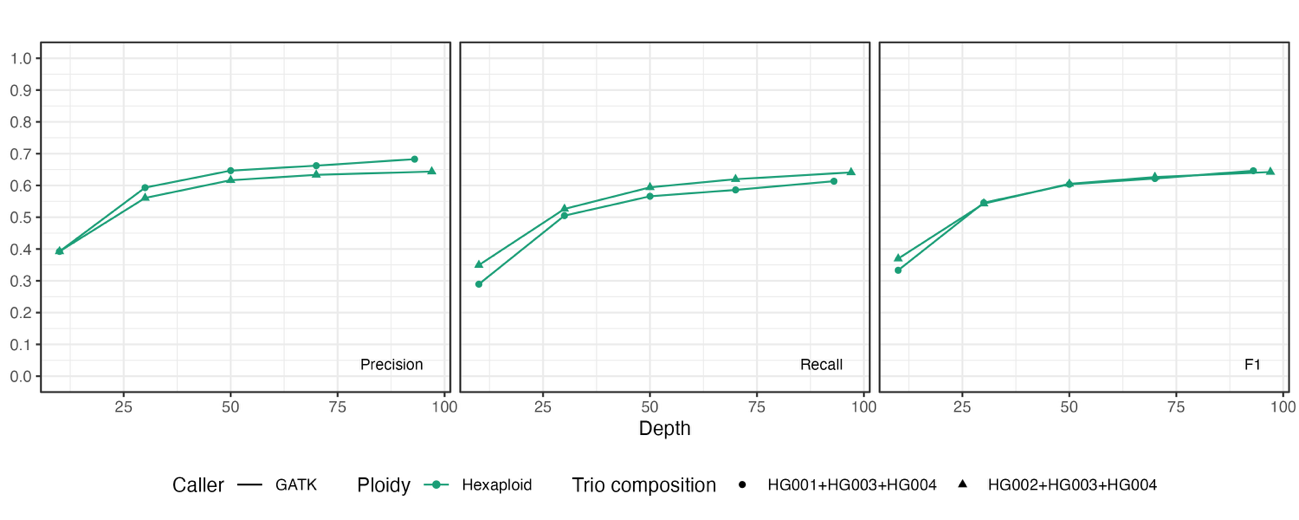


Figure S32 Performance of indel genotyping by GATK on chromosome 20 of the synthetic human hexaploid genomes using high-accuracy long reads. Precision, recall, and F1 scores are presented
