# Supplementary material for: Benchmarking long-read variant calling in diploid and polyploid genomes: insights from human and plants
Source: BMC Genomics. 2026 Jan 15;27:46. doi: 10.1186/s12864-025-12259-5 (PMC12809965; doi:10.1186/s12864-025-12259-5)
Supplement: Supplementary file 2 — Supplementary Material 2. [file 12864_2025_12259_MOESM2_ESM.docx]

**Supplementary Tables**

**Table S1. Accession IDs or links for PacBio HiFi datasets**

| Species | Identifier | Source | Data id/link |
| --- | --- | --- | --- |
| *Homo sapiens* | HG001 | NCBI | SRR9001768 |
|  |  |  | SRR9001769 |
|  |  |  | SRR9001770 |
|  |  |  | SRR9001771 |
|  |  |  | SRR9001772 |
|  |  |  | SRR9001773 |
| *Homo sapiens* | HG002 | HPRC | https://s3-us-west-2.amazonaws.com/human-pangenomics/index.html?prefix=NHGRI_UCSC_panel/HG002/hpp_HG002_NA24385_son_v1/PacBio_HiFi/downsampled/15kb/ |
| *Homo sapiens* | HG003 | HPRC | https://s3-us-west-2.amazonaws.com/human-pangenomics/index.html?prefix=NHGRI_UCSC_panel/HG003/PacBio_HiFi/ |
| *Homo sapiens* | HG004 | HPRC | https://s3-us-west-2.amazonaws.com/human-pangenomics/index.html?prefix=NHGRI_UCSC_panel/HG004/PacBio_HiFi/ |
| *Fragaria vesca* | drFraVesc1 | EBI | ERR12954112 |
| *Solanum tuberosum* | Otava | NCBI | SRR15206231 |
| *Zea mays* | Mo17 | NCBI | SRR15447414 |
|  |  |  | SRR15447415 |
|  |  |  | SRR15447416 |
|  |  |  | SRR15447418 |
|  |  |  | SRR15447419 |
|  |  |  | SRR15447420 |
|  |  |  | SRR15447421 |

**Table S2. Mapping statistics for all datasets**

| ***H. sapiens*** | | | ***F. vesca*** | | | ***S. tuberosum*** | | | ***Z. mays*** | | |
| --- | --- | --- | --- | --- | --- | --- | --- | --- | --- | --- | --- |
| MAPQ | Count | Fraction | MAPQ | Count | Fraction | MAPQ | Count | Fraction | MAPQ | Count | Fraction |
| 0 | 8458 | 0.00101407 | 0 | 350 | 0.09322792 | 0 | 1639 | 0.05120309 | 0 | 137872 | 0.03414924 |
| 1 | 259113 | 0.0310664 | 1 | 5849 | 1.55797179 | 1 | 222641 | 6.95540333 | 1 | 579858 | 0.14362388 |
| 2 | 14491 | 0.0017374 | 2 | 423 | 0.1126726 | 2 | 7876 | 0.24604972 | 2 | 28456 | 0.00704821 |
| 3 | 12640 | 0.00151547 | 3 | 358 | 0.09535885 | 3 | 7657 | 0.23920807 | 3 | 27346 | 0.00677328 |
| 4 | 11023 | 0.0013216 | 4 | 321 | 0.08550332 | 4 | 7179 | 0.22427514 | 4 | 24431 | 0.00605127 |
| 5 | 10009 | 0.00120003 | 5 | 290 | 0.07724599 | 5 | 7421 | 0.23183532 | 5 | 23003 | 0.00569757 |
| 6 | 8924 | 0.00106994 | 6 | 262 | 0.06978776 | 6 | 7052 | 0.2203076 | 6 | 21523 | 0.00533099 |
| 7 | 8056 | 0.00096588 | 7 | 248 | 0.06605864 | 7 | 6698 | 0.20924848 | 7 | 20314 | 0.00503153 |
| 8 | 7235 | 0.00086744 | 8 | 196 | 0.05220764 | 8 | 6673 | 0.20846747 | 8 | 19647 | 0.00486633 |
| 9 | 6645 | 0.0007967 | 9 | 161 | 0.04288484 | 9 | 6426 | 0.20075108 | 9 | 19116 | 0.0047348 |
| 10 | 6041 | 0.00072429 | 10 | 175 | 0.04661396 | 10 | 6287 | 0.19640866 | 10 | 18585 | 0.00460328 |
| 11 | 5604 | 0.00067189 | 11 | 165 | 0.04395031 | 11 | 6211 | 0.19403439 | 11 | 17778 | 0.0044034 |
| 12 | 5270 | 0.00063185 | 12 | 139 | 0.0370248 | 12 | 6109 | 0.19084786 | 12 | 16996 | 0.00420971 |
| 13 | 5131 | 0.00061518 | 13 | 150 | 0.03995482 | 13 | 5943 | 0.18566195 | 13 | 16523 | 0.00409255 |
| 14 | 4857 | 0.00058233 | 14 | 89 | 0.02370653 | 14 | 5912 | 0.1846935 | 14 | 15862 | 0.00392883 |
| 15 | 4751 | 0.00056962 | 15 | 90 | 0.02397289 | 15 | 5874 | 0.18350636 | 15 | 15233 | 0.00377303 |
| 16 | 4540 | 0.00054432 | 16 | 140 | 0.03729117 | 16 | 5726 | 0.17888277 | 16 | 14778 | 0.00366033 |
| 17 | 4234 | 0.00050764 | 17 | 99 | 0.02637018 | 17 | 5754 | 0.17975751 | 17 | 14288 | 0.00353897 |
| 18 | 3871 | 0.00046411 | 18 | 134 | 0.03569298 | 18 | 5670 | 0.17713331 | 18 | 13976 | 0.00346169 |
| 19 | 3673 | 0.00044037 | 19 | 75 | 0.01997741 | 19 | 5677 | 0.17735199 | 19 | 13288 | 0.00329128 |
| 20 | 3314 | 0.00039733 | 20 | 75 | 0.01997741 | 20 | 5511 | 0.17216608 | 20 | 12925 | 0.00320137 |
| 21 | 3264 | 0.00039134 | 21 | 125 | 0.03329569 | 21 | 5421 | 0.16935444 | 21 | 12749 | 0.00315777 |
| 22 | 2967 | 0.00035573 | 22 | 104 | 0.02770201 | 22 | 5358 | 0.16738629 | 22 | 12319 | 0.00305127 |
| 23 | 2852 | 0.00034194 | 23 | 68 | 0.01811285 | 23 | 5097 | 0.15923253 | 23 | 11767 | 0.00291454 |
| 24 | 2838 | 0.00034026 | 24 | 85 | 0.02264107 | 24 | 5046 | 0.15763927 | 24 | 11287 | 0.00279565 |
| 25 | 2745 | 0.00032911 | 25 | 76 | 0.02024378 | 25 | 4989 | 0.15585857 | 25 | 11011 | 0.00272729 |
| 26 | 2699 | 0.0003236 | 26 | 67 | 0.01784649 | 26 | 5322 | 0.16626163 | 26 | 10752 | 0.00266314 |
| 27 | 2647 | 0.00031736 | 27 | 62 | 0.01651466 | 27 | 5045 | 0.15760803 | 27 | 10402 | 0.00257645 |
| 28 | 2506 | 0.00030046 | 28 | 62 | 0.01651466 | 28 | 4986 | 0.15576485 | 28 | 10054 | 0.00249026 |
| 29 | 2383 | 0.00028571 | 29 | 130 | 0.03462751 | 29 | 4891 | 0.152797 | 29 | 9908 | 0.00245409 |
| 30 | 2454 | 0.00029422 | 30 | 62 | 0.01651466 | 30 | 4947 | 0.15454647 | 30 | 9511 | 0.00235576 |
| 31 | 2375 | 0.00028475 | 31 | 48 | 0.01278554 | 31 | 4985 | 0.15573361 | 31 | 9267 | 0.00229532 |
| 32 | 2345 | 0.00028115 | 32 | 70 | 0.01864558 | 32 | 4995 | 0.15604601 | 32 | 9017 | 0.0022334 |
| 33 | 2250 | 0.00026976 | 33 | 63 | 0.01678103 | 33 | 4762 | 0.14876699 | 33 | 8765 | 0.00217099 |
| 34 | 2331 | 0.00027948 | 34 | 55 | 0.0146501 | 34 | 4755 | 0.1485483 | 34 | 8709 | 0.00215711 |
| 35 | 2321 | 0.00027828 | 35 | 52 | 0.01385101 | 35 | 4735 | 0.14792349 | 35 | 8239 | 0.0020407 |
| 36 | 2195 | 0.00026317 | 36 | 569 | 0.15156197 | 36 | 4763 | 0.14879823 | 36 | 8295 | 0.00205457 |
| 37 | 2305 | 0.00027636 | 37 | 79 | 0.02104287 | 37 | 4611 | 0.14404968 | 37 | 7976 | 0.00197556 |
| 38 | 2220 | 0.00026617 | 38 | 74 | 0.01971105 | 38 | 4566 | 0.14264386 | 38 | 7566 | 0.00187401 |
| 39 | 2202 | 0.00026401 | 39 | 62 | 0.01651466 | 39 | 4616 | 0.14420588 | 39 | 7536 | 0.00186658 |
| 40 | 2136 | 0.0002561 | 40 | 36 | 0.00958916 | 40 | 4372 | 0.13658321 | 40 | 7365 | 0.00182422 |
| 41 | 2116 | 0.0002537 | 41 | 119 | 0.03169749 | 41 | 4446 | 0.13889501 | 41 | 7188 | 0.00178038 |
| 42 | 2124 | 0.00025466 | 42 | 68 | 0.01811285 | 42 | 4380 | 0.13683314 | 42 | 7103 | 0.00175933 |
| 43 | 2091 | 0.0002507 | 43 | 62 | 0.01651466 | 43 | 4225 | 0.13199087 | 43 | 6831 | 0.00169196 |
| 44 | 2174 | 0.00026065 | 44 | 56 | 0.01491647 | 44 | 4365 | 0.13636453 | 44 | 6809 | 0.00168651 |
| 45 | 2004 | 0.00024027 | 45 | 67 | 0.01784649 | 45 | 4306 | 0.13452134 | 45 | 6597 | 0.001634 |
| 46 | 2047 | 0.00024543 | 46 | 53 | 0.01411737 | 46 | 4114 | 0.12852318 | 46 | 6427 | 0.00159189 |
| 47 | 2119 | 0.00025406 | 47 | 50 | 0.01331827 | 47 | 4160 | 0.12996024 | 47 | 6375 | 0.00157901 |
| 48 | 2026 | 0.00024291 | 48 | 62 | 0.01651466 | 48 | 4183 | 0.13067877 | 48 | 6216 | 0.00153963 |
| 49 | 2040 | 0.00024459 | 49 | 67 | 0.01784649 | 49 | 4088 | 0.12771093 | 49 | 6009 | 0.00148836 |
| 50 | 1924 | 0.00023068 | 50 | 84 | 0.0223747 | 50 | 4174 | 0.13039761 | 50 | 6086 | 0.00150743 |
| 51 | 1988 | 0.00023835 | 51 | 44 | 0.01172008 | 51 | 4066 | 0.12702364 | 51 | 6102 | 0.00151139 |
| 52 | 1969 | 0.00023607 | 52 | 72 | 0.01917832 | 52 | 4200 | 0.13120986 | 52 | 5883 | 0.00145715 |
| 53 | 1894 | 0.00022708 | 53 | 68 | 0.01811285 | 53 | 4100 | 0.12808581 | 53 | 5828 | 0.00144353 |
| 54 | 1985 | 0.00023799 | 54 | 116 | 0.0308984 | 54 | 4233 | 0.13224079 | 54 | 5660 | 0.00140191 |
| 55 | 1957 | 0.00023463 | 55 | 51 | 0.01358464 | 55 | 4089 | 0.12774217 | 55 | 5649 | 0.00139919 |
| 56 | 1954 | 0.00023428 | 56 | 58 | 0.0154492 | 56 | 5462 | 0.1706353 | 56 | 5778 | 0.00143114 |
| 57 | 1990 | 0.00023859 | 57 | 64 | 0.01704739 | 57 | 4025 | 0.12574278 | 57 | 5550 | 0.00137467 |
| 58 | 2044 | 0.00024507 | 58 | 147 | 0.03915573 | 58 | 3964 | 0.12383711 | 58 | 5602 | 0.00138755 |
| 59 | 2048 | 0.00024555 | 59 | 56 | 0.01491647 | 59 | 4051 | 0.12655503 | 59 | 5580 | 0.0013821 |
| 60 | 7854211 | 0.94168191 | 60 | 362322 | 96.5100793 | 60 | 2676150 | 83.6041099 | 60 | 2655771 | 0.65780266 |
|  | Total reads | 8341915 |  | Total reads | 383165 |  | Total reads | 3203243 |  | Total reads | 4038836 |
|  | # mapped | 8340620 |  | # mapped | 375424 |  | # mapped | 3200979 |  | # mapped | 4037337 |
|  | # unmapped | 1295 |  | # unmapped | 7741 |  | # unmapped | 2264 |  | # unmapped | 1499 |
|  | % mapped | 0.99984476 |  | % mapped | 0.97979722 |  | % mapped | 0.99929322 |  | % mapped | 0.99962885 |

Mapping statistics for the datasets used in this study, including *Homo sapiens* (HG002), *Fragaria vesca* (drFraVesc1), *Solanum tuberosum* (Otava), and *Zea mays* (Mo17). The BAM file with the highest sequencing depth for each respective sample was selected for quality validation.

**Table S3. Performance metrics across species and callers**

| **GATK (All)** | depth | ploidy | Type | True-pos-baseline | True-pos-call | False-pos | False-neg | Precision | Sensitivity | F-measure |
| --- | --- | --- | --- | --- | --- | --- | --- | --- | --- | --- |
| *H. sapiens* | 10 | 2 | Genotyping | 3699426 | 3721639 | 107122 | 191098 | 0.972 | 0.9509 | 0.9613 |
| *H. sapiens* | 32 | 2 | Genotyping | 3827291 | 3857904 | 62606 | 63233 | 0.984 | 0.9837 | 0.9839 |
| *H. sapiens* | 10 | 4 | Genotyping | 3188863 | 3203214 | 1233217 | 1821149 | 0.722 | 0.6365 | 0.6766 |
| *H. sapiens* | 30 | 4 | Genotyping | 4405119 | 4441149 | 555804 | 604711 | 0.8888 | 0.8793 | 0.884 |
| *H. sapiens* | 64 | 4 | Genotyping | 4718837 | 4760346 | 286233 | 290918 | 0.9433 | 0.9419 | 0.9426 |
| *H. sapiens* | 10 | 6 | Genotyping | 2407647 | 2413776 | 1876210 | 2538076 | 0.5627 | 0.4868 | 0.522 |
| *H. sapiens* | 30 | 6 | Genotyping | 3651235 | 3668324 | 1165183 | 1287380 | 0.7589 | 0.7393 | 0.749 |
| *H. sapiens* | 50 | 6 | Genotyping | 4065455 | 4088162 | 819548 | 870646 | 0.833 | 0.8236 | 0.8283 |
| *H. sapiens* | 70 | 6 | Genotyping | 4292253 | 4319325 | 623181 | 642585 | 0.8739 | 0.8698 | 0.8718 |
| *H. sapiens* | 97 | 6 | Genotyping | 4463260 | 4494342 | 469986 | 470627 | 0.9053 | 0.9046 | 0.905 |
| *F. vesca* | 10 | 2 | Genotyping | 260833 | 260805 | 40842 | 28427 | 0.8646 | 0.9017 | 0.8828 |
| *F. vesca* | 30 | 2 | Genotyping | 259482 | 259511 | 44037 | 29778 | 0.8549 | 0.8971 | 0.8755 |
| *S. tuberosum* | 10 | 4 | Genotyping | 1489822 | 1481463 | 1363225 | 2461022 | 0.5208 | 0.3771 | 0.4374 |
| *S. tuberosum* | 30 | 4 | Genotyping | 2255167 | 2242803 | 1155795 | 1691312 | 0.6599 | 0.5714 | 0.6125 |
| *S. tuberosum* | 60 | 4 | Genotyping | 2436126 | 2422913 | 1015336 | 1507286 | 0.7047 | 0.6178 | 0.6584 |
| *Z. mays* | 10 | 2 | Genotyping | 2184570 | 2183675 | 1991655 | 648388 | 0.523 | 0.7711 | 0.6233 |
| *Z. mays* | 30 | 2 | Genotyping | 1990989 | 1990196 | 2664696 | 841968 | 0.4275 | 0.7028 | 0.5317 |
| **GATK (SNV)** | depth | ploidy | Type | True-pos-baseline | True-pos-call | False-pos | False-neg | Precision | Sensitivity | F-measure |
| *H. sapiens* | 10 | 2 | Genotyping | 3268264 | 3269740 | 21496 | 96851 | 0.9935 | 0.9712 | 0.9822 |
| *H. sapiens* | 32 | 2 | Genotyping | 3355135 | 3356848 | 6101 | 9980 | 0.9982 | 0.997 | 0.9976 |
| *H. sapiens* | 10 | 4 | Genotyping | 2901169 | 2902619 | 964245 | 1509600 | 0.7506 | 0.6577 | 0.7011 |
| *H. sapiens* | 30 | 4 | Genotyping | 4002956 | 4005740 | 369088 | 407724 | 0.9156 | 0.9076 | 0.9116 |
| *H. sapiens* | 64 | 4 | Genotyping | 4289500 | 4292510 | 110805 | 121133 | 0.9748 | 0.9725 | 0.9737 |
| *H. sapiens* | 10 | 6 | Genotyping | 2195103 | 2195844 | 1566684 | 2181357 | 0.5836 | 0.5016 | 0.5395 |
| *H. sapiens* | 30 | 6 | Genotyping | 3343355 | 3345277 | 926496 | 1032424 | 0.7831 | 0.7641 | 0.7735 |
| *H. sapiens* | 50 | 6 | Genotyping | 3728325 | 3730580 | 594605 | 647292 | 0.8625 | 0.8521 | 0.8573 |
| *H. sapiens* | 70 | 6 | Genotyping | 3940597 | 3943013 | 402653 | 434953 | 0.9073 | 0.9006 | 0.904 |
| *H. sapiens* | 97 | 6 | Genotyping | 4101711 | 4104259 | 252021 | 273788 | 0.9421 | 0.9374 | 0.9398 |
| *F. vesca* | 10 | 2 | Genotyping | 222220 | 220219 | 14563 | 11002 | 0.938 | 0.9528 | 0.9453 |
| *F. vesca* | 30 | 2 | Genotyping | 224427 | 222639 | 19214 | 8795 | 0.9206 | 0.9623 | 0.941 |
| *S. tuberosum* | 10 | 4 | Genotyping | 1365198 | 1340812 | 1183937 | 2175661 | 0.5311 | 0.3856 | 0.4468 |
| *S. tuberosum* | 30 | 4 | Genotyping | 2064885 | 2026048 | 989707 | 1474030 | 0.6718 | 0.5835 | 0.6245 |
| *S. tuberosum* | 60 | 4 | Genotyping | 2228003 | 2185334 | 861565 | 1308573 | 0.7172 | 0.63 | 0.6708 |
| *Z. mays* | 10 | 2 | Genotyping | 2017967 | 2007561 | 1742975 | 567099 | 0.5353 | 0.7806 | 0.6351 |
| *Z. mays* | 30 | 2 | Genotyping | 1839696 | 1830734 | 2344435 | 745369 | 0.4385 | 0.7117 | 0.5426 |
| **GATK (Indel)** | depth | ploidy | Type | True-pos-baseline | True-pos-call | False-pos | False-neg | Precision | Sensitivity | F-measure |
| *H. sapiens* | 10 | 2 | Genotyping | 431162 | 451899 | 85626 | 94247 | 0.8407 | 0.8206 | 0.8305 |
| *H. sapiens* | 32 | 2 | Genotyping | 472156 | 501056 | 56505 | 53253 | 0.8987 | 0.8986 | 0.8987 |
| *H. sapiens* | 10 | 4 | Genotyping | 287694 | 300595 | 268972 | 311549 | 0.5278 | 0.4801 | 0.5028 |
| *H. sapiens* | 30 | 4 | Genotyping | 402163 | 435409 | 186716 | 196987 | 0.6999 | 0.6712 | 0.6852 |
| *H. sapiens* | 64 | 4 | Genotyping | 429337 | 467836 | 175428 | 169785 | 0.7273 | 0.7166 | 0.7219 |
| *H. sapiens* | 10 | 6 | Genotyping | 212544 | 217932 | 309526 | 356719 | 0.4132 | 0.3734 | 0.3923 |
| *H. sapiens* | 30 | 6 | Genotyping | 307880 | 323047 | 238687 | 254956 | 0.5751 | 0.547 | 0.5607 |
| *H. sapiens* | 50 | 6 | Genotyping | 337130 | 357582 | 224943 | 223354 | 0.6138 | 0.6015 | 0.6076 |
| *H. sapiens* | 70 | 6 | Genotyping | 351656 | 376312 | 220528 | 207632 | 0.6305 | 0.6288 | 0.6296 |
| *H. sapiens* | 97 | 6 | Genotyping | 361549 | 390083 | 217965 | 196839 | 0.6415 | 0.6475 | 0.6445 |
| *F. vesca* | 10 | 2 | Genotyping | 38613 | 40586 | 26279 | 17425 | 0.607 | 0.6891 | 0.6454 |
| *F. vesca* | 30 | 2 | Genotyping | 35055 | 36872 | 24823 | 20983 | 0.5976 | 0.6256 | 0.6113 |
| *S. tuberosum* | 10 | 4 | Genotyping | 124624 | 140651 | 179288 | 285361 | 0.4396 | 0.304 | 0.3594 |
| *S. tuberosum* | 30 | 4 | Genotyping | 190282 | 216755 | 166088 | 217282 | 0.5662 | 0.4669 | 0.5118 |
| *S. tuberosum* | 60 | 4 | Genotyping | 208123 | 237579 | 153771 | 198713 | 0.6071 | 0.5116 | 0.5552 |
| *Z. mays* | 10 | 2 | Genotyping | 166603 | 176114 | 248680 | 81289 | 0.4146 | 0.6721 | 0.5128 |
| *Z. mays* | 30 | 2 | Genotyping | 151293 | 159462 | 320261 | 96599 | 0.3324 | 0.6103 | 0.4304 |
| **GATK (All)** | depth | ploidy | Type | True-pos-baseline | True-pos-call | False-pos | False-neg | Precision | Sensitivity | F-measure |
| *H. sapiens* | 10 | 1 | Allele detection | 3758240 | 3758300 | 70461 | 132284 | 0.9816 | 0.966 | 0.9737 |
| *H. sapiens* | 32 | 1 | Allele detection | 3867199 | 3867146 | 53364 | 23325 | 0.9864 | 0.994 | 0.9902 |
| *H. sapiens* | 10 | 1 | Allele detection | 4318959 | 4322753 | 113863 | 691229 | 0.9743 | 0.862 | 0.9148 |
| *H. sapiens* | 30 | 1 | Allele detection | 4885159 | 4898785 | 98633 | 125029 | 0.9803 | 0.975 | 0.9776 |
| *H. sapiens* | 64 | 1 | Allele detection | 4923490 | 4942343 | 104783 | 86698 | 0.9792 | 0.9827 | 0.981 |
| *H. sapiens* | 10 | 1 | Allele detection | 4198281 | 4201617 | 99304 | 753088 | 0.9769 | 0.8479 | 0.9078 |
| *H. sapiens* | 30 | 1 | Allele detection | 4757584 | 4773347 | 93701 | 193785 | 0.9807 | 0.9609 | 0.9707 |
| *H. sapiens* | 50 | 1 | Allele detection | 4817966 | 4842780 | 107728 | 133403 | 0.9782 | 0.9731 | 0.9756 |
| *H. sapiens* | 70 | 1 | Allele detection | 4841109 | 4872384 | 117684 | 110260 | 0.9764 | 0.9777 | 0.9771 |
| *H. sapiens* | 97 | 1 | Allele detection | 4853386 | 4890067 | 125414 | 97983 | 0.975 | 0.9802 | 0.9776 |
| *F. vesca* | 10 | 1 | Allele detection | 268067 | 268021 | 33626 | 21193 | 0.8885 | 0.9267 | 0.9072 |
| *F. vesca* | 30 | 1 | Allele detection | 270959 | 270924 | 32624 | 18301 | 0.8925 | 0.9367 | 0.9141 |
| *S. tuberosum* | 10 | 1 | Allele detection | 2650283 | 2623703 | 239943 | 1334560 | 0.9162 | 0.6651 | 0.7707 |
| *S. tuberosum* | 30 | 1 | Allele detection | 3108332 | 3076691 | 345986 | 876383 | 0.8989 | 0.7801 | 0.8353 |
| *S. tuberosum* | 60 | 1 | Allele detection | 3140983 | 3108971 | 355686 | 843715 | 0.8973 | 0.7883 | 0.8393 |
| *Z. mays* | 10 | 1 | Allele detection | 2616072 | 2615147 | 1560222 | 216886 | 0.6263 | 0.9234 | 0.7464 |
| *Z. mays* | 30 | 1 | Allele detection | 2669011 | 2668099 | 1986850 | 163947 | 0.5732 | 0.9421 | 0.7127 |
| **GATK (SNV)** | depth | ploidy | Type | True-pos-baseline | True-pos-call | False-pos | False-neg | Precision | Sensitivity | F-measure |
| *H. sapiens* | 10 | 1 | Allele detection | 3286664 | 3286926 | 4310 | 78451 | 0.9987 | 0.9767 | 0.9876 |
| *H. sapiens* | 32 | 1 | Allele detection | 3358273 | 3358471 | 4478 | 6842 | 0.9987 | 0.998 | 0.9983 |
| *H. sapiens* | 10 | 1 | Allele detection | 3860668 | 3861119 | 5828 | 550196 | 0.9985 | 0.8753 | 0.9328 |
| *H. sapiens* | 30 | 1 | Allele detection | 4365859 | 4366290 | 8724 | 45005 | 0.998 | 0.9898 | 0.9939 |
| *H. sapiens* | 64 | 1 | Allele detection | 4394971 | 4395392 | 8143 | 15893 | 0.9982 | 0.9964 | 0.9973 |
| *H. sapiens* | 10 | 1 | Allele detection | 3759153 | 3759690 | 4464 | 618825 | 0.9988 | 0.8587 | 0.9234 |
| *H. sapiens* | 30 | 1 | Allele detection | 4266880 | 4267514 | 6678 | 111098 | 0.9984 | 0.9746 | 0.9864 |
| *H. sapiens* | 50 | 1 | Allele detection | 4320792 | 4321398 | 6407 | 57186 | 0.9985 | 0.9869 | 0.9927 |
| *H. sapiens* | 70 | 1 | Allele detection | 4341249 | 4341839 | 6520 | 36729 | 0.9985 | 0.9916 | 0.995 |
| *H. sapiens* | 97 | 1 | Allele detection | 4351975 | 4352540 | 6469 | 26003 | 0.9985 | 0.9941 | 0.9963 |
| *F. vesca* | 10 | 1 | Allele detection | 224454 | 222274 | 12508 | 8768 | 0.9467 | 0.9624 | 0.9545 |
| *F. vesca* | 30 | 1 | Allele detection | 228202 | 226020 | 15833 | 5020 | 0.9345 | 0.9785 | 0.956 |
| *S. tuberosum* | 10 | 1 | Allele detection | 2410883 | 2344941 | 192230 | 1157379 | 0.9242 | 0.6756 | 0.7806 |
| *S. tuberosum* | 30 | 1 | Allele detection | 2829327 | 2750465 | 278659 | 738831 | 0.908 | 0.7929 | 0.8466 |
| *S. tuberosum* | 60 | 1 | Allele detection | 2857695 | 2777509 | 283945 | 710451 | 0.9073 | 0.8009 | 0.8508 |
| *Z. mays* | 10 | 1 | Allele detection | 2388501 | 2388501 | 1362056 | 196565 | 0.6368 | 0.924 | 0.754 |
| *Z. mays* | 30 | 1 | Allele detection | 2429340 | 2429340 | 1745866 | 155726 | 0.5818 | 0.9398 | 0.7187 |
| **GATK (Indel)** | depth | ploidy | Type | True-pos-baseline | True-pos-call | False-pos | False-neg | Precision | Sensitivity | F-measure |
| *H. sapiens* | 10 | 1 | Allele detection | 471576 | 471374 | 66151 | 53833 | 0.8769 | 0.8975 | 0.8871 |
| *H. sapiens* | 32 | 1 | Allele detection | 508926 | 508675 | 48886 | 16483 | 0.9123 | 0.9686 | 0.9396 |
| *H. sapiens* | 10 | 1 | Allele detection | 458291 | 461634 | 108035 | 141033 | 0.8104 | 0.7647 | 0.7869 |
| *H. sapiens* | 30 | 1 | Allele detection | 519300 | 532495 | 89909 | 80024 | 0.8555 | 0.8665 | 0.861 |
| *H. sapiens* | 64 | 1 | Allele detection | 528519 | 546951 | 96640 | 70805 | 0.8498 | 0.8819 | 0.8656 |
| *H. sapiens* | 10 | 1 | Allele detection | 439128 | 441927 | 94840 | 134263 | 0.8233 | 0.7658 | 0.7935 |
| *H. sapiens* | 30 | 1 | Allele detection | 490704 | 505833 | 87023 | 82687 | 0.8532 | 0.8558 | 0.8545 |
| *H. sapiens* | 50 | 1 | Allele detection | 497174 | 521382 | 101321 | 76217 | 0.8373 | 0.8671 | 0.8519 |
| *H. sapiens* | 70 | 1 | Allele detection | 499860 | 530545 | 111164 | 73531 | 0.8268 | 0.8718 | 0.8487 |
| *H. sapiens* | 97 | 1 | Allele detection | 501411 | 537527 | 118945 | 71980 | 0.8188 | 0.8745 | 0.8457 |
| *F. vesca* | 10 | 1 | Allele detection | 43613 | 45747 | 21118 | 12425 | 0.6842 | 0.7783 | 0.7282 |
| *F. vesca* | 30 | 1 | Allele detection | 42757 | 44904 | 16791 | 13281 | 0.7278 | 0.763 | 0.745 |
| *S. tuberosum* | 10 | 1 | Allele detection | 239400 | 278762 | 47713 | 177181 | 0.8539 | 0.5747 | 0.687 |
| *S. tuberosum* | 30 | 1 | Allele detection | 279005 | 326226 | 67327 | 137552 | 0.8289 | 0.6698 | 0.7409 |
| *S. tuberosum* | 60 | 1 | Allele detection | 283288 | 331462 | 71741 | 133264 | 0.8221 | 0.6801 | 0.7444 |
| *Z. mays* | 10 | 1 | Allele detection | 213672 | 225006 | 199806 | 34220 | 0.5297 | 0.862 | 0.6561 |
| *Z. mays* | 30 | 1 | Allele detection | 225183 | 237156 | 242587 | 22709 | 0.4943 | 0.9084 | 0.6403 |
| **FreeBayes** |  |  |  |  |  |  |  |  |  |  |
| **FreeBayes (All)** | depth | ploidy | Type | True-pos-baseline | True-pos-call | False-pos | False-neg | Precision | Sensitivity | F-measure |
| *H. sapiens* | 10 | 2 | Genotyping | 3400950 | 3429163 | 385311 | 489530 | 0.899 | 0.8742 | 0.8864 |
| *H. sapiens* | 32 | 2 | Genotyping | 3746735 | 3785320 | 343589 | 143729 | 0.9168 | 0.9631 | 0.9394 |
| *H. sapiens* | 10 | 4 | Genotyping | 2784685 | 2801425 | 1675316 | 2370266 | 0.6258 | 0.5402 | 0.5798 |
| *H. sapiens* | 30 | 4 | Genotyping | 4257315 | 4289615 | 1482570 | 897364 | 0.7432 | 0.8259 | 0.7824 |
| *H. sapiens* | 64 | 4 | Genotyping | 4661171 | 4699037 | 1096754 | 493302 | 0.8108 | 0.9043 | 0.855 |
| *H. sapiens* | 10 | 6 | Genotyping | 2178677 | 2186783 | 2273549 | 3015130 | 0.4903 | 0.4195 | 0.4521 |
| *H. sapiens* | 30 | 6 | Genotyping | 3587160 | 3609047 | 2677180 | 1600739 | 0.5741 | 0.6914 | 0.6273 |
| *H. sapiens* | 50 | 6 | Genotyping | 4106732 | 4133661 | 2513863 | 1079734 | 0.6218 | 0.7918 | 0.6966 |
| *H. sapiens* | 70 | 6 | Genotyping | 4359597 | 4389813 | 2220049 | 826072 | 0.6641 | 0.8407 | 0.7421 |
| *H. sapiens* | 97 | 6 | Genotyping | 4531678 | 4564186 | 1979873 | 653217 | 0.6975 | 0.874 | 0.7758 |
| *F. vesca* | 10 | 2 | Genotyping | 235933 | 236562 | 95321 | 52882 | 0.7128 | 0.8169 | 0.7613 |
| *F. vesca* | 30 | 2 | Genotyping | 247655 | 248279 | 123281 | 41293 | 0.6682 | 0.8571 | 0.751 |
| *S. tuberosum* | 10 | 4 | Genotyping | 1594513 | 1598370 | 2668140 | 2332157 | 0.3746 | 0.4061 | 0.3897 |
| *S. tuberosum* | 30 | 4 | Genotyping | 2526981 | 2534529 | 3719689 | 1356927 | 0.4053 | 0.6506 | 0.4994 |
| *S. tuberosum* | 60 | 4 | Genotyping | 2783533 | 2792170 | 3962648 | 1093143 | 0.4134 | 0.718 | 0.5247 |
| *Z. mays* | 10 | 2 | Genotyping | 1851845 | 1857305 | 7988912 | 980565 | 0.1886 | 0.6538 | 0.2928 |
| *Z. mays* | 30 | 2 | Genotyping | 1836643 | 1853202 | 9381444 | 995737 | 0.165 | 0.6484 | 0.263 |
| **FreeBayes (SNV)** | depth | ploidy | Type | True-pos-baseline | True-pos-call | False-pos | False-neg | Precision | Sensitivity | F-measure |
| *H. sapiens* | 10 | 2 | Genotyping | 3028829 | 3036932 | 64265 | 336278 | 0.9793 | 0.9001 | 0.938 |
| *H. sapiens* | 32 | 2 | Genotyping | 3317482 | 3328794 | 58234 | 47622 | 0.9828 | 0.9858 | 0.9843 |
| *H. sapiens* | 10 | 4 | Genotyping | 2523678 | 2527388 | 857287 | 1973430 | 0.7467 | 0.5612 | 0.6408 |
| *H. sapiens* | 30 | 4 | Genotyping | 3879539 | 3886095 | 408869 | 617405 | 0.9048 | 0.8627 | 0.8833 |
| *H. sapiens* | 64 | 4 | Genotyping | 4238745 | 4246409 | 153057 | 258058 | 0.9652 | 0.9426 | 0.9538 |
| *H. sapiens* | 10 | 6 | Genotyping | 1982655 | 1984474 | 1360072 | 2533535 | 0.5933 | 0.439 | 0.5046 |
| *H. sapiens* | 30 | 6 | Genotyping | 3278296 | 3282482 | 964396 | 1236846 | 0.7729 | 0.7261 | 0.7488 |
| *H. sapiens* | 50 | 6 | Genotyping | 3747707 | 3752883 | 620051 | 767443 | 0.8582 | 0.83 | 0.8439 |
| *H. sapiens* | 70 | 6 | Genotyping | 3975292 | 3981066 | 426420 | 539865 | 0.9033 | 0.8804 | 0.8917 |
| *H. sapiens* | 97 | 6 | Genotyping | 4130962 | 4137061 | 278738 | 384055 | 0.9369 | 0.9149 | 0.9258 |
| *F. vesca* | 10 | 2 | Genotyping | 206907 | 207543 | 66232 | 25945 | 0.7581 | 0.8886 | 0.8182 |
| *F. vesca* | 30 | 2 | Genotyping | 218206 | 218835 | 85327 | 14775 | 0.7195 | 0.9366 | 0.8138 |
| *S. tuberosum* | 10 | 4 | Genotyping | 1477791 | 1482229 | 2512663 | 2044823 | 0.371 | 0.4195 | 0.3938 |
| *S. tuberosum* | 30 | 4 | Genotyping | 2338419 | 2346656 | 3539370 | 1150646 | 0.3987 | 0.6702 | 0.5 |
| *S. tuberosum* | 60 | 4 | Genotyping | 2572951 | 2582241 | 3787367 | 910831 | 0.4054 | 0.7386 | 0.5235 |
| *Z. mays* | 10 | 2 | Genotyping | 1740254 | 1745904 | 7476896 | 844415 | 0.1893 | 0.6733 | 0.2955 |
| *Z. mays* | 30 | 2 | Genotyping | 1731375 | 1748335 | 8822875 | 853279 | 0.1654 | 0.6699 | 0.2653 |
| **FreeBayes (Indel)** | depth | ploidy | Type | True-pos-baseline | True-pos-call | False-pos | False-neg | Precision | Sensitivity | F-measure |
| *H. sapiens* | 10 | 2 | Genotyping | 372121 | 392231 | 321046 | 153252 | 0.5499 | 0.7083 | 0.6191 |
| *H. sapiens* | 32 | 2 | Genotyping | 429253 | 456526 | 285355 | 96107 | 0.6154 | 0.8171 | 0.702 |
| *H. sapiens* | 10 | 4 | Genotyping | 261007 | 274037 | 818029 | 396836 | 0.2509 | 0.3968 | 0.3074 |
| *H. sapiens* | 30 | 4 | Genotyping | 377776 | 403520 | 1073701 | 279959 | 0.2732 | 0.5744 | 0.3702 |
| *H. sapiens* | 64 | 4 | Genotyping | 422426 | 452628 | 943697 | 235244 | 0.3242 | 0.6423 | 0.4309 |
| *H. sapiens* | 10 | 6 | Genotyping | 196022 | 202309 | 913477 | 481595 | 0.1813 | 0.2893 | 0.2229 |
| *H. sapiens* | 30 | 6 | Genotyping | 308864 | 326565 | 1712784 | 363893 | 0.1601 | 0.4591 | 0.2374 |
| *H. sapiens* | 50 | 6 | Genotyping | 359025 | 380778 | 1893812 | 312291 | 0.1674 | 0.5348 | 0.255 |
| *H. sapiens* | 70 | 6 | Genotyping | 384305 | 408747 | 1793629 | 286207 | 0.1856 | 0.5732 | 0.2804 |
| *H. sapiens* | 97 | 6 | Genotyping | 400716 | 427125 | 1701135 | 269162 | 0.2007 | 0.5982 | 0.3006 |
| *F. vesca* | 10 | 2 | Genotyping | 29026 | 29019 | 29089 | 26937 | 0.4994 | 0.5187 | 0.5088 |
| *F. vesca* | 30 | 2 | Genotyping | 29449 | 29444 | 37954 | 26518 | 0.4369 | 0.5262 | 0.4774 |
| *S. tuberosum* | 10 | 4 | Genotyping | 116722 | 116141 | 155477 | 287334 | 0.4276 | 0.2889 | 0.3448 |
| *S. tuberosum* | 30 | 4 | Genotyping | 188562 | 187873 | 180319 | 206281 | 0.5103 | 0.4776 | 0.4934 |
| *S. tuberosum* | 60 | 4 | Genotyping | 210582 | 209929 | 175281 | 182312 | 0.545 | 0.536 | 0.5404 |
| *Z. mays* | 10 | 2 | Genotyping | 111591 | 111401 | 512016 | 136150 | 0.1787 | 0.4504 | 0.2559 |
| *Z. mays* | 30 | 2 | Genotyping | 105268 | 104867 | 558569 | 142458 | 0.1581 | 0.4249 | 0.2304 |
| **FreeBayes (All)** | depth | ploidy | Type | True-pos-baseline | True-pos-call | False-pos | False-neg | Precision | Sensitivity | F-measure |
| *H. sapiens* | 10 | 2 | Allele detection | 3465570 | 3472284 | 343086 | 424954 | 0.9101 | 0.8908 | 0.9003 |
| *H. sapiens* | 32 | 2 | Allele detection | 3803707 | 3812786 | 317224 | 86817 | 0.9232 | 0.9777 | 0.9497 |
| *H. sapiens* | 10 | 4 | Allele detection | 3752264 | 3760518 | 718587 | 1403089 | 0.8396 | 0.7278 | 0.7797 |
| *H. sapiens* | 30 | 4 | Allele detection | 4691127 | 4706931 | 1070753 | 464226 | 0.8147 | 0.91 | 0.8597 |
| *H. sapiens* | 64 | 4 | Allele detection | 4820534 | 4838864 | 961766 | 334819 | 0.8342 | 0.9351 | 0.8818 |
| *H. sapiens* | 10 | 6 | Allele detection | 3707587 | 3715625 | 761880 | 1491937 | 0.8298 | 0.7131 | 0.767 |
| *H. sapiens* | 30 | 6 | Allele detection | 4638768 | 4659762 | 1665329 | 560756 | 0.7367 | 0.8922 | 0.807 |
| *H. sapiens* | 50 | 6 | Allele detection | 4795475 | 4819995 | 1871535 | 404049 | 0.7203 | 0.9223 | 0.8089 |
| *H. sapiens* | 70 | 6 | Allele detection | 4842244 | 4869038 | 1787372 | 357280 | 0.7315 | 0.9313 | 0.8194 |
| *H. sapiens* | 97 | 6 | Allele detection | 4857345 | 4886502 | 1706573 | 342179 | 0.7412 | 0.9342 | 0.8266 |
| *F. vesca* | 10 | 2 | Allele detection | 244329 | 245028 | 87692 | 44931 | 0.7364 | 0.8447 | 0.7868 |
| *F. vesca* | 30 | 2 | Allele detection | 260984 | 261828 | 111698 | 28276 | 0.701 | 0.9022 | 0.789 |
| *S. tuberosum* | 10 | 4 | Allele detection | 2497107 | 2534866 | 1962390 | 1486138 | 0.5636 | 0.6269 | 0.5936 |
| *S. tuberosum* | 30 | 4 | Allele detection | 3235014 | 3383227 | 3534776 | 745549 | 0.489 | 0.8127 | 0.6106 |
| *S. tuberosum* | 60 | 4 | Allele detection | 3355351 | 3563143 | 3823618 | 620474 | 0.4824 | 0.8439 | 0.6139 |
| *Z. mays* | 10 | 2 | Allele detection | 2278356 | 2289269 | 7592587 | 554562 | 0.2317 | 0.8042 | 0.3597 |
| *Z. mays* | 30 | 2 | Allele detection | 2277112 | 2304586 | 8970059 | 555837 | 0.2044 | 0.8038 | 0.3259 |
| **FreeBayes (SNV)** | depth | ploidy | Type | True-pos-baseline | True-pos-call | False-pos | False-neg | Precision | Sensitivity | F-measure |
| *H. sapiens* | 10 | 2 | Allele detection | 3047531 | 3052952 | 44431 | 317584 | 0.9857 | 0.9056 | 0.9439 |
| *H. sapiens* | 32 | 2 | Allele detection | 3325028 | 3333755 | 49954 | 40087 | 0.9852 | 0.9881 | 0.9867 |
| *H. sapiens* | 10 | 4 | Allele detection | 3338288 | 3340523 | 41144 | 1158976 | 0.9878 | 0.7423 | 0.8476 |
| *H. sapiens* | 30 | 4 | Allele detection | 4218628 | 4219263 | 71178 | 278636 | 0.9834 | 0.938 | 0.9602 |
| *H. sapiens* | 64 | 4 | Allele detection | 4327756 | 4331062 | 65093 | 169508 | 0.9852 | 0.9623 | 0.9736 |
| *H. sapiens* | 10 | 6 | Allele detection | 3301895 | 3303038 | 42536 | 1215568 | 0.9873 | 0.7309 | 0.84 |
| *H. sapiens* | 30 | 6 | Allele detection | 4163859 | 4159625 | 84413 | 353604 | 0.9801 | 0.9217 | 0.95 |
| *H. sapiens* | 50 | 6 | Allele detection | 4294826 | 4292868 | 79574 | 222637 | 0.9818 | 0.9507 | 0.966 |
| *H. sapiens* | 70 | 6 | Allele detection | 4331578 | 4330895 | 76560 | 185885 | 0.9826 | 0.9589 | 0.9706 |
| *H. sapiens* | 97 | 6 | Allele detection | 4342160 | 4342843 | 73975 | 175303 | 0.9833 | 0.9612 | 0.9721 |
| *F. vesca* | 10 | 2 | Allele detection | 210207 | 210922 | 63633 | 23015 | 0.7682 | 0.9013 | 0.8295 |
| *F. vesca* | 30 | 2 | Allele detection | 222300 | 223173 | 82892 | 10922 | 0.7292 | 0.9532 | 0.8263 |
| *S. tuberosum* | 10 | 4 | Allele detection | 2305538 | 2345607 | 1874425 | 1261318 | 0.5558 | 0.6464 | 0.5977 |
| *S. tuberosum* | 30 | 4 | Allele detection | 2990707 | 3142776 | 3396922 | 573925 | 0.4806 | 0.839 | 0.6111 |
| *S. tuberosum* | 60 | 4 | Allele detection | 3100014 | 3312172 | 3678540 | 460371 | 0.4738 | 0.8707 | 0.6137 |
| *Z. mays* | 10 | 2 | Allele detection | 2115638 | 2114448 | 7081044 | 469393 | 0.2299 | 0.8184 | 0.359 |
| *Z. mays* | 30 | 2 | Allele detection | 2113486 | 2129980 | 8443967 | 471574 | 0.2014 | 0.8176 | 0.3232 |
| **FreeBayes (Indel)** | depth | ploidy | Type | True-pos-baseline | True-pos-call | False-pos | False-neg | Precision | Sensitivity | F-measure |
| *H. sapiens* | 10 | 2 | Allele detection | 418039 | 419332 | 298655 | 107370 | 0.584 | 0.7956 | 0.6736 |
| *H. sapiens* | 32 | 2 | Allele detection | 478679 | 479031 | 267270 | 46730 | 0.6419 | 0.9111 | 0.7531 |
| *H. sapiens* | 10 | 4 | Allele detection | 413976 | 419995 | 677443 | 244113 | 0.3827 | 0.6291 | 0.4759 |
| *H. sapiens* | 30 | 4 | Allele detection | 472499 | 487668 | 999575 | 185590 | 0.3279 | 0.718 | 0.4502 |
| *H. sapiens* | 64 | 4 | Allele detection | 492778 | 507802 | 896673 | 165311 | 0.3616 | 0.7488 | 0.4877 |
| *H. sapiens* | 10 | 6 | Allele detection | 405692 | 412587 | 719344 | 276369 | 0.3645 | 0.5948 | 0.452 |
| *H. sapiens* | 30 | 6 | Allele detection | 474909 | 500137 | 1580916 | 207152 | 0.2403 | 0.6963 | 0.3573 |
| *H. sapiens* | 50 | 6 | Allele detection | 500649 | 527127 | 1791961 | 181412 | 0.2273 | 0.734 | 0.3471 |
| *H. sapiens* | 70 | 6 | Allele detection | 510666 | 538143 | 1710812 | 171395 | 0.2393 | 0.7487 | 0.3627 |
| *H. sapiens* | 97 | 6 | Allele detection | 515185 | 543659 | 1632598 | 166876 | 0.2498 | 0.7553 | 0.3755 |
| *F. vesca* | 10 | 2 | Allele detection | 34122 | 34106 | 24059 | 21916 | 0.5864 | 0.6089 | 0.5974 |
| *F. vesca* | 30 | 2 | Allele detection | 38684 | 38655 | 28806 | 17354 | 0.573 | 0.6903 | 0.6262 |
| *S. tuberosum* | 10 | 4 | Allele detection | 191569 | 189259 | 87965 | 224820 | 0.6827 | 0.4601 | 0.5497 |
| *S. tuberosum* | 30 | 4 | Allele detection | 244307 | 240451 | 137854 | 171624 | 0.6356 | 0.5874 | 0.6105 |
| *S. tuberosum* | 60 | 4 | Allele detection | 255337 | 250971 | 145078 | 160103 | 0.6337 | 0.6146 | 0.624 |
| *Z. mays* | 10 | 2 | Allele detection | 162718 | 174821 | 511543 | 85169 | 0.2547 | 0.6564 | 0.367 |
| *Z. mays* | 30 | 2 | Allele detection | 163626 | 174606 | 526092 | 84263 | 0.2492 | 0.6601 | 0.3618 |

Each row corresponds to a species, and each column reports evaluation metrics including precision, recall, and F1 score for all, SNVs and indels. Results are shown for each variant caller and ploidy configuration where applicable. Benchmarking was performed using the corresponding truth set for each genome. All performance values reflect post-filtered variants within confident regions. See Methods for benchmarking criteria.

**Table S4. Coding-region subset metrics in synthetic human polyploids**

| **GATK (All)** | depth | ploidy | Type | True-pos-baseline | True-pos-call | False-pos | False-neg | Precision | Sensitivity | F-measure |
| --- | --- | --- | --- | --- | --- | --- | --- | --- | --- | --- |
| *H. sapiens* | 10 | 4 | Genotyping | 20821 | 20852 | 8935 | 13585 | 0.7 | 0.6052 | 0.6491 |
| *H. sapiens* | 30 | 4 | Genotyping | 30428 | 30495 | 3634 | 3978 | 0.8935 | 0.8844 | 0.8889 |
| *H. sapiens* | 64 | 4 | Genotyping | 33004 | 33075 | 1338 | 1402 | 0.9611 | 0.9593 | 0.9602 |
| *H. sapiens* | 10 | 6 | Genotyping | 16139 | 16154 | 12965 | 18023 | 0.5548 | 0.4724 | 0.5103 |
| *H. sapiens* | 30 | 6 | Genotyping | 25152 | 25190 | 7969 | 9007 | 0.7597 | 0.7363 | 0.7478 |
| *H. sapiens* | 50 | 6 | Genotyping | 28340 | 28388 | 5237 | 5816 | 0.8443 | 0.8297 | 0.8369 |
| *H. sapiens* | 70 | 6 | Genotyping | 30042 | 30096 | 3767 | 4113 | 0.8888 | 0.8796 | 0.8841 |
| *H. sapiens* | 97 | 6 | Genotyping | 31548 | 31611 | 2441 | 2603 | 0.9283 | 0.9238 | 0.926 |
| **GATK (SNV)** | depth | ploidy | Type | True-pos-baseline | True-pos-call | False-pos | False-neg | Precision | Sensitivity | F-measure |
| *H. sapiens* | 10 | 4 | Genotyping | 20212 | 20230 | 7657 | 12974 | 0.7254 | 0.6091 | 0.6622 |
| *H. sapiens* | 30 | 4 | Genotyping | 29533 | 29558 | 3232 | 3653 | 0.9014 | 0.8899 | 0.8956 |
| *H. sapiens* | 64 | 4 | Genotyping | 32046 | 32074 | 1086 | 1140 | 0.9672 | 0.9656 | 0.9664 |
| *H. sapiens* | 10 | 6 | Genotyping | 15639 | 15646 | 11741 | 17337 | 0.5713 | 0.4743 | 0.5183 |
| *H. sapiens* | 30 | 6 | Genotyping | 24467 | 24493 | 7432 | 8508 | 0.7672 | 0.742 | 0.7544 |
| *H. sapiens* | 50 | 6 | Genotyping | 27553 | 27572 | 4840 | 5421 | 0.8507 | 0.8356 | 0.8431 |
| *H. sapiens* | 70 | 6 | Genotyping | 29220 | 29240 | 3425 | 3754 | 0.8951 | 0.8862 | 0.8906 |
| *H. sapiens* | 97 | 6 | Genotyping | 30680 | 30703 | 2119 | 2290 | 0.9354 | 0.9305 | 0.933 |
| **GATK (Indel)** | depth | ploidy | Type | True-pos-baseline | True-pos-call | False-pos | False-neg | Precision | Sensitivity | F-measure |
| *H. sapiens* | 10 | 4 | Genotyping | 609 | 622 | 1278 | 611 | 0.3274 | 0.4992 | 0.3954 |
| *H. sapiens* | 30 | 4 | Genotyping | 895 | 937 | 402 | 325 | 0.6998 | 0.7336 | 0.7163 |
| *H. sapiens* | 64 | 4 | Genotyping | 958 | 1001 | 252 | 262 | 0.7989 | 0.7852 | 0.792 |
| *H. sapiens* | 10 | 6 | Genotyping | 500 | 508 | 1224 | 686 | 0.2933 | 0.4216 | 0.3459 |
| *H. sapiens* | 30 | 6 | Genotyping | 685 | 697 | 537 | 499 | 0.5648 | 0.5785 | 0.5716 |
| *H. sapiens* | 50 | 6 | Genotyping | 787 | 816 | 397 | 395 | 0.6727 | 0.6658 | 0.6692 |
| *H. sapiens* | 70 | 6 | Genotyping | 822 | 856 | 342 | 359 | 0.7145 | 0.696 | 0.7052 |
| *H. sapiens* | 97 | 6 | Genotyping | 868 | 908 | 322 | 313 | 0.7382 | 0.735 | 0.7366 |
| **GATK (All)** | depth | ploidy | Type | True-pos-baseline | True-pos-call | False-pos | False-neg | Precision | Sensitivity | F-measure |
| *H. sapiens* | 10 | 4 | Allele detection | 28760 | 28761 | 1029 | 5651 | 0.9655 | 0.8358 | 0.896 |
| *H. sapiens* | 30 | 4 | Allele detection | 33820 | 33828 | 304 | 591 | 0.9911 | 0.9828 | 0.9869 |
| *H. sapiens* | 64 | 4 | Allele detection | 34214 | 34221 | 195 | 197 | 0.9943 | 0.9943 | 0.9943 |
| *H. sapiens* | 10 | 6 | Allele detection | 28294 | 28293 | 848 | 5885 | 0.9709 | 0.8278 | 0.8937 |
| *H. sapiens* | 30 | 6 | Allele detection | 32926 | 32936 | 248 | 1253 | 0.9925 | 0.9633 | 0.9777 |
| *H. sapiens* | 50 | 6 | Allele detection | 33442 | 33460 | 198 | 737 | 0.9941 | 0.9784 | 0.9862 |
| *H. sapiens* | 70 | 6 | Allele detection | 33684 | 33703 | 197 | 495 | 0.9942 | 0.9855 | 0.9898 |
| *H. sapiens* | 97 | 6 | Allele detection | 33860 | 33881 | 215 | 319 | 0.9937 | 0.9907 | 0.9922 |
| **GATK (SNV)** | depth | ploidy | Type | True-pos-baseline | True-pos-call | False-pos | False-neg | Precision | Sensitivity | F-measure |
| *H. sapiens* | 10 | 4 | Allele detection | 27826 | 27820 | 70 | 5365 | 0.9975 | 0.8384 | 0.911 |
| *H. sapiens* | 30 | 4 | Allele detection | 32718 | 32709 | 84 | 473 | 0.9974 | 0.9857 | 0.9916 |
| *H. sapiens* | 64 | 4 | Allele detection | 33097 | 33088 | 75 | 94 | 0.9977 | 0.9972 | 0.9975 |
| *H. sapiens* | 10 | 6 | Allele detection | 27380 | 27372 | 30 | 5611 | 0.9989 | 0.8299 | 0.9066 |
| *H. sapiens* | 30 | 6 | Allele detection | 31891 | 31890 | 48 | 1100 | 0.9985 | 0.9667 | 0.9823 |
| *H. sapiens* | 50 | 6 | Allele detection | 32384 | 32380 | 48 | 607 | 0.9985 | 0.9816 | 0.99 |
| *H. sapiens* | 70 | 6 | Allele detection | 32628 | 32618 | 61 | 363 | 0.9981 | 0.989 | 0.9935 |
| *H. sapiens* | 97 | 6 | Allele detection | 32791 | 32782 | 61 | 200 | 0.9981 | 0.9939 | 0.996 |
| **GATK (Indel)** | depth | ploidy | Type | True-pos-baseline | True-pos-call | False-pos | False-neg | Precision | Sensitivity | F-measure |
| *H. sapiens* | 10 | 4 | Allele detection | 934 | 941 | 959 | 286 | 0.4953 | 0.7656 | 0.6014 |
| *H. sapiens* | 30 | 4 | Allele detection | 1102 | 1119 | 220 | 118 | 0.8357 | 0.9033 | 0.8682 |
| *H. sapiens* | 64 | 4 | Allele detection | 1117 | 1133 | 120 | 103 | 0.9042 | 0.9156 | 0.9099 |
| *H. sapiens* | 10 | 6 | Allele detection | 914 | 921 | 818 | 274 | 0.5296 | 0.7694 | 0.6274 |
| *H. sapiens* | 30 | 6 | Allele detection | 1035 | 1046 | 200 | 153 | 0.8395 | 0.8712 | 0.8551 |
| *H. sapiens* | 50 | 6 | Allele detection | 1058 | 1080 | 150 | 130 | 0.878 | 0.8906 | 0.8843 |
| *H. sapiens* | 70 | 6 | Allele detection | 1056 | 1085 | 136 | 132 | 0.8886 | 0.8889 | 0.8888 |
| *H. sapiens* | 97 | 6 | Allele detection | 1069 | 1099 | 154 | 119 | 0.8771 | 0.8998 | 0.8883 |
| **FreeBayes (All)** | depth | ploidy | Type | True-pos-baseline | True-pos-call | False-pos | False-neg | Precision | Sensitivity | F-measure |
| *H. sapiens* | 10 | 4 | Genotyping | 17942 | 17713 | 13705 | 16469 | 0.5638 | 0.5214 | 0.5418 |
| *H. sapiens* | 30 | 4 | Genotyping | 29419 | 28965 | 14544 | 4992 | 0.6657 | 0.8549 | 0.7486 |
| *H. sapiens* | 64 | 4 | Genotyping | 32886 | 32377 | 10646 | 1525 | 0.7526 | 0.9557 | 0.842 |
| *H. sapiens* | 10 | 6 | Genotyping | 14399 | 14200 | 18138 | 19769 | 0.4391 | 0.4214 | 0.4301 |
| *H. sapiens* | 30 | 6 | Genotyping | 24298 | 23985 | 27624 | 9866 | 0.4647 | 0.7112 | 0.5622 |
| *H. sapiens* | 50 | 6 | Genotyping | 28335 | 27949 | 26932 | 5829 | 0.5093 | 0.8294 | 0.631 |
| *H. sapiens* | 70 | 6 | Genotyping | 30306 | 29878 | 24597 | 3858 | 0.5485 | 0.8871 | 0.6778 |
| *H. sapiens* | 97 | 6 | Genotyping | 31703 | 31252 | 21539 | 2455 | 0.592 | 0.9281 | 0.7229 |
| **FreeBayes (SNV)** | depth | ploidy | Type | True-pos-baseline | True-pos-call | False-pos | False-neg | Precision | Sensitivity | F-measure |
| *H. sapiens* | 10 | 4 | Genotyping | 17398 | 16935 | 6125 | 15793 | 0.7344 | 0.5242 | 0.6117 |
| *H. sapiens* | 30 | 4 | Genotyping | 28587 | 27352 | 2850 | 4604 | 0.9056 | 0.8613 | 0.8829 |
| *H. sapiens* | 64 | 4 | Genotyping | 31929 | 30709 | 980 | 1262 | 0.9691 | 0.962 | 0.9655 |
| *H. sapiens* | 10 | 6 | Genotyping | 13990 | 13593 | 9367 | 18990 | 0.592 | 0.4242 | 0.4943 |
| *H. sapiens* | 30 | 6 | Genotyping | 23648 | 22759 | 6726 | 9328 | 0.7719 | 0.7171 | 0.7435 |
| *H. sapiens* | 50 | 6 | Genotyping | 27571 | 26597 | 4297 | 5405 | 0.8609 | 0.8361 | 0.8483 |
| *H. sapiens* | 70 | 6 | Genotyping | 29476 | 28484 | 2991 | 3500 | 0.905 | 0.8939 | 0.8994 |
| *H. sapiens* | 97 | 6 | Genotyping | 30841 | 29828 | 1905 | 2129 | 0.94 | 0.9354 | 0.9377 |
| **FreeBayes (Indel)** | depth | ploidy | Type | True-pos-baseline | True-pos-call | False-pos | False-neg | Precision | Sensitivity | F-measure |
| *H. sapiens* | 10 | 4 | Genotyping | 544 | 778 | 7580 | 676 | 0.0931 | 0.4459 | 0.154 |
| *H. sapiens* | 30 | 4 | Genotyping | 832 | 1613 | 11694 | 388 | 0.1212 | 0.682 | 0.2058 |
| *H. sapiens* | 64 | 4 | Genotyping | 957 | 1668 | 9666 | 263 | 0.1472 | 0.7844 | 0.2478 |
| *H. sapiens* | 10 | 6 | Genotyping | 409 | 607 | 8771 | 779 | 0.0647 | 0.3443 | 0.109 |
| *H. sapiens* | 30 | 6 | Genotyping | 650 | 1226 | 20898 | 538 | 0.0554 | 0.5471 | 0.1006 |
| *H. sapiens* | 50 | 6 | Genotyping | 764 | 1352 | 22635 | 424 | 0.0564 | 0.6431 | 0.1036 |
| *H. sapiens* | 70 | 6 | Genotyping | 830 | 1394 | 21606 | 358 | 0.0606 | 0.6987 | 0.1115 |
| *H. sapiens* | 97 | 6 | Genotyping | 862 | 1424 | 19634 | 326 | 0.0676 | 0.7256 | 0.1237 |
| **FreeBayes (All)** | depth | ploidy | Type | True-pos-baseline | True-pos-call | False-pos | False-neg | Precision | Sensitivity | F-measure |
| *H. sapiens* | 10 | 4 | Allele detection | 24661 | 24295 | 6854 | 9750 | 0.78 | 0.7167 | 0.747 |
| *H. sapiens* | 30 | 4 | Allele detection | 32793 | 32300 | 10908 | 1618 | 0.7475 | 0.953 | 0.8379 |
| *H. sapiens* | 64 | 4 | Allele detection | 34013 | 33510 | 9258 | 398 | 0.7835 | 0.9884 | 0.8741 |
| *H. sapiens* | 10 | 6 | Allele detection | 24606 | 24246 | 7785 | 9573 | 0.757 | 0.7199 | 0.738 |
| *H. sapiens* | 30 | 6 | Allele detection | 32052 | 31600 | 19613 | 2127 | 0.617 | 0.9378 | 0.7443 |
| *H. sapiens* | 50 | 6 | Allele detection | 33354 | 32878 | 21623 | 825 | 0.6033 | 0.9759 | 0.7456 |
| *H. sapiens* | 70 | 6 | Allele detection | 33756 | 33273 | 20834 | 423 | 0.6149 | 0.9876 | 0.758 |
| *H. sapiens* | 97 | 6 | Allele detection | 33922 | 33440 | 18984 | 257 | 0.6379 | 0.9925 | 0.7766 |
| **FreeBayes (SNV)** | depth | ploidy | Type | True-pos-baseline | True-pos-call | False-pos | False-neg | Precision | Sensitivity | F-measure |
| *H. sapiens* | 10 | 4 | Allele detection | 23810 | 22979 | 81 | 9381 | 0.9965 | 0.7174 | 0.8342 |
| *H. sapiens* | 30 | 4 | Allele detection | 31748 | 30037 | 165 | 1443 | 0.9945 | 0.9565 | 0.9752 |
| *H. sapiens* | 64 | 4 | Allele detection | 32919 | 31479 | 210 | 272 | 0.9934 | 0.9918 | 0.9926 |
| *H. sapiens* | 10 | 6 | Allele detection | 23776 | 22944 | 16 | 9215 | 0.9993 | 0.7207 | 0.8374 |
| *H. sapiens* | 30 | 6 | Allele detection | 31036 | 29292 | 193 | 1955 | 0.9935 | 0.9407 | 0.9664 |
| *H. sapiens* | 50 | 6 | Allele detection | 32278 | 30697 | 197 | 713 | 0.9936 | 0.9784 | 0.9859 |
| *H. sapiens* | 70 | 6 | Allele detection | 32656 | 31236 | 239 | 335 | 0.9924 | 0.9898 | 0.9911 |
| *H. sapiens* | 97 | 6 | Allele detection | 32813 | 31499 | 234 | 178 | 0.9926 | 0.9946 | 0.9936 |
| **FreeBayes (Indel)** | depth | ploidy | Type | True-pos-baseline | True-pos-call | False-pos | False-neg | Precision | Sensitivity | F-measure |
| *H. sapiens* | 10 | 4 | Allele detection | 851 | 1316 | 6773 | 369 | 0.1627 | 0.6975 | 0.2638 |
| *H. sapiens* | 30 | 4 | Allele detection | 1045 | 2263 | 10743 | 175 | 0.174 | 0.8566 | 0.2892 |
| *H. sapiens* | 64 | 4 | Allele detection | 1094 | 2031 | 9048 | 126 | 0.1833 | 0.8967 | 0.3044 |
| *H. sapiens* | 10 | 6 | Allele detection | 830 | 1302 | 7769 | 358 | 0.1435 | 0.6987 | 0.2381 |
| *H. sapiens* | 30 | 6 | Allele detection | 1016 | 2308 | 19420 | 172 | 0.1062 | 0.8552 | 0.189 |
| *H. sapiens* | 50 | 6 | Allele detection | 1076 | 2181 | 21426 | 112 | 0.0924 | 0.9057 | 0.1677 |
| *H. sapiens* | 70 | 6 | Allele detection | 1100 | 2037 | 20595 | 88 | 0.09 | 0.9259 | 0.1641 |
| *H. sapiens* | 97 | 6 | Allele detection | 1109 | 1941 | 18750 | 79 | 0.0938 | 0.9335 | 0.1705 |

Performance metrics for small-variant detection across all synthetic human polyploids and all variant callers evaluated in this study. Each column reports evaluation metrics, including precision, recall, and F1 score, for all variants, SNVs, and indels. Results are shown for each variant caller and ploidy configuration where applicable. Benchmarking was performed based on overlap between coding regions and the corresponding truth set for the human polyploids. All performance values reflect post-filtered variants within confident regions. See Methods for benchmarking criteria.

**Table S5. True vs false-positive indel densities (hexaploid, 70×, FreeBayes)**

|  | True indels/1000 | False-positive indels/1000 |
| --- | --- | --- |
| Coding | 0.034489574 | 0.972667222 |
| All confident | 0.233318975 | 1.600540074 |

Occurrence of true indels and false-positive indels at 70× hexaploidy using FreeBayes. Numbers are per 1000 bp.

**Table S6. Effect of MQM and QD on FreeBayes (HG002 chr1)**.

| MQM | Depth | Category | Precision | Sensitivity | F-measure |
| --- | --- | --- | --- | --- | --- |
| 1 | 10 | MQM1-Depth10 | 0.9013 | 0.8776 | 0.8893 |
| 1 | 32 | MQM1-Depth32 | 0.9202 | **0.9665** | **0.9428** |
| 60 | 10 | MQM60-Depth10 | 0.9025 | 0.874 | 0.888 |
| 60 | 32 | MQM60-Depth32 | 0.9227 | 0.9586 | 0.9403 |
| 1 | 10 | MQM1-Depth10 with GATK-like filter | 0.936 | 0.8454 | 0.8884 |
| 1 | 32 | MQM1-Depth32 with GATK-like filter | 0.9383 | 0.906 | 0.9218 |
| 60 | 10 | MQM60-Depth10 with GATK-like filter | 0.9369 | 0.842 | 0.8869 |
| 60 | 32 | MQM60-Depth32 with GATK-like filter | **0.9401** | 0.8987 | 0.9189 |

**Table S7. Effect of MQM and QD on FreeBayes (synthetic hexaploid chr1)**

| MQM | Depth | Category | Precision | Sensitivity | F-measure |
| --- | --- | --- | --- | --- | --- |
| 1 | 97 | MQM1-Depth97 (ploidy: 6) | 0.7033 | 0.9002 | 0.7897 |
| 60 | 97 | MQM60-Depth97 (ploidy: 6) | 0.7035 | 0.8888 | 0.7854 |
| 1 | 97 | MQM1-Depth97 with GATK-like filter (ploidy: 6) | **0.766** | **0.8698** | **0.8156** |
| 60 | 97 | MQM60-Depth97 with GATK-like filter (ploidy: 6) | **0.766** | 0.8588 | 0.8097 |

**Table S8. FreeBayes under stricter filtering in tetra/hexaploids**

| **FreeBayes (All)** | depth | ploidy | Type | True-pos-baseline | True-pos-call | False-pos | False-neg | Precision | Sensitivity | F-measure |
| --- | --- | --- | --- | --- | --- | --- | --- | --- | --- | --- |
| *H. sapiens* | 10 | 4 | Genotyping | 53707 | 53489 | 27838 | 53747 | 0.6577 | 0.4998 | 0.568 |
| *H. sapiens* | 30 | 4 | Genotyping | 86143 | 85755 | 21287 | 21311 | 0.8011 | 0.8017 | 0.8014 |
| *H. sapiens* | 64 | 4 | Genotyping | 95684 | 95248 | 15158 | 11770 | 0.8627 | 0.8905 | 0.8764 |
| *H. sapiens* | 10 | 6 | Genotyping | 41168 | 41051 | 39825 | 65141 | 0.5076 | 0.3872 | 0.4393 |
| *H. sapiens* | 30 | 6 | Genotyping | 70387 | 70169 | 40424 | 35777 | 0.6345 | 0.663 | 0.6484 |
| *H. sapiens* | 50 | 6 | Genotyping | 82303 | 82039 | 35326 | 23785 | 0.699 | 0.7758 | 0.7354 |
| *H. sapiens* | 70 | 6 | Genotyping | 87906 | 87593 | 31957 | 18157 | 0.7327 | 0.8288 | 0.7778 |
| *H. sapiens* | 97 | 6 | Genotyping | 92062 | 91727 | 28169 | 13981 | 0.7651 | 0.8682 | 0.8134 |
| *S. tuberosum* | 60 | 4 | Genotyping | 2134504 | 1940167 | 1195358 | 1818811 | 0.6188 | 0.5399 | 0.5767 |
| *S. tuberosum* | 30 | 4 | Genotyping | 1916019 | 1747750 | 1210238 | 2039894 | 0.5909 | 0.4843 | 0.5323 |
| *S. tuberosum* | 10 | 4 | Genotyping | 1225083 | 1139212 | 1147245 | 2740219 | 0.4982 | 0.309 | 0.3814 |
| **FreeBayes (SNV)** | depth | ploidy | Type | True-pos-baseline | True-pos-call | False-pos | False-neg | Precision | Sensitivity | F-measure |
| *H. sapiens* | 10 | 4 | Genotyping | 48787 | 48608 | 17596 | 45914 | 0.7342 | 0.5152 | 0.6055 |
| *H. sapiens* | 30 | 4 | Genotyping | 78587 | 78272 | 6605 | 16114 | 0.9222 | 0.8298 | 0.8736 |
| *H. sapiens* | 64 | 4 | Genotyping | 87147 | 86793 | 1781 | 7554 | 0.9799 | 0.9202 | 0.9491 |
| *H. sapiens* | 10 | 6 | Genotyping | 37487 | 37386 | 27790 | 56679 | 0.5736 | 0.3981 | 0.47 |
| *H. sapiens* | 30 | 6 | Genotyping | 64826 | 64645 | 17993 | 29330 | 0.7823 | 0.6885 | 0.7324 |
| *H. sapiens* | 50 | 6 | Genotyping | 75659 | 75434 | 11285 | 18493 | 0.8699 | 0.8036 | 0.8354 |
| *H. sapiens* | 70 | 6 | Genotyping | 80714 | 80444 | 7687 | 13438 | 0.9128 | 0.8573 | 0.8842 |
| *H. sapiens* | 97 | 6 | Genotyping | 84538 | 84261 | 4501 | 9620 | 0.9493 | 0.8978 | 0.9228 |
| *S. tuberosum* | 60 | 4 | Genotyping | 1869530 | 1701410 | 940979 | 1674801 | 0.6439 | 0.5275 | 0.5799 |
| *S. tuberosum* | 30 | 4 | Genotyping | 1682741 | 1536377 | 951211 | 1863535 | 0.6176 | 0.4745 | 0.5367 |
| *S. tuberosum* | 10 | 4 | Genotyping | 1086755 | 1010336 | 933495 | 2466485 | 0.5198 | 0.3058 | 0.3851 |
| **FreeBayes (Indel)** | depth | ploidy | Type | True-pos-baseline | True-pos-call | False-pos | False-neg | Precision | Sensitivity | F-measure |
| *H. sapiens* | 10 | 4 | Genotyping | 4920 | 4881 | 10242 | 7833 | 0.3228 | 0.3858 | 0.3515 |
| *H. sapiens* | 30 | 4 | Genotyping | 7556 | 7483 | 14682 | 5197 | 0.3376 | 0.5925 | 0.4301 |
| *H. sapiens* | 64 | 4 | Genotyping | 8537 | 8455 | 13377 | 4216 | 0.3873 | 0.6694 | 0.4907 |
| *H. sapiens* | 10 | 6 | Genotyping | 3681 | 3665 | 12035 | 8462 | 0.2334 | 0.3031 | 0.2638 |
| *H. sapiens* | 30 | 6 | Genotyping | 5561 | 5524 | 22431 | 6447 | 0.1976 | 0.4631 | 0.277 |
| *H. sapiens* | 50 | 6 | Genotyping | 6644 | 6605 | 24041 | 5292 | 0.2155 | 0.5566 | 0.3107 |
| *H. sapiens* | 70 | 6 | Genotyping | 7192 | 7149 | 24270 | 4719 | 0.2275 | 0.6038 | 0.3305 |
| *H. sapiens* | 97 | 6 | Genotyping | 7524 | 7466 | 23668 | 4361 | 0.2398 | 0.6331 | 0.3478 |
| *S. tuberosum* | 60 | 4 | Genotyping | 264974 | 238757 | 254379 | 144010 | 0.4842 | 0.6479 | 0.5542 |
| *S. tuberosum* | 30 | 4 | Genotyping | 233278 | 211373 | 259027 | 176359 | 0.4493 | 0.5695 | 0.5023 |
| *S. tuberosum* | 10 | 4 | Genotyping | 138328 | 128876 | 213750 | 273734 | 0.3761 | 0.3357 | 0.3548 |
| **FreeBayes (All)** | depth | ploidy | Type | True-pos-baseline | True-pos-call | False-pos | False-neg | Precision | Sensitivity | F-measure |
| *H. sapiens* | 10 | 4 | Allele detection | 75576 | 75407 | 5920 | 31878 | 0.9272 | 0.7033 | 0.7999 |
| *H. sapiens* | 30 | 4 | Allele detection | 96853 | 96682 | 10358 | 10601 | 0.9032 | 0.9013 | 0.9023 |
| *H. sapiens* | 64 | 4 | Allele detection | 100398 | 100278 | 10128 | 7056 | 0.9083 | 0.9343 | 0.9211 |
| *H. sapiens* | 10 | 6 | Allele detection | 74170 | 74028 | 6954 | 32205 | 0.9141 | 0.6973 | 0.7911 |
| *H. sapiens* | 30 | 6 | Allele detection | 94659 | 94578 | 16347 | 11716 | 0.8526 | 0.8899 | 0.8708 |
| *H. sapiens* | 50 | 6 | Allele detection | 98741 | 98708 | 19124 | 7634 | 0.8377 | 0.9282 | 0.8806 |
| *H. sapiens* | 70 | 6 | Allele detection | 100226 | 100255 | 19806 | 6149 | 0.835 | 0.9422 | 0.8854 |
| *H. sapiens* | 97 | 6 | Allele detection | 100862 | 100929 | 19535 | 5513 | 0.8378 | 0.9482 | 0.8896 |
| *S. tuberosum* | 60 | 1 | Allele detection | 2553149 | 2314173 | 825705 | 1431697 | 0.737 | 0.6407 | 0.6855 |
| *S. tuberosum* | 30 | 1 | Allele detection | 2460429 | 2232552 | 729352 | 1524417 | 0.7538 | 0.6174 | 0.6788 |
| *S. tuberosum* | 10 | 1 | Allele detection | 1951081 | 1795106 | 493555 | 2033765 | 0.7843 | 0.4896 | 0.6029 |
| **FreeBayes (SNV)** | depth | ploidy | Type | True-pos-baseline | True-pos-call | False-pos | False-neg | Precision | Sensitivity | F-measure |
| *H. sapiens* | 10 | 4 | Allele detection | 66908 | 66179 | 25 | 27793 | 0.9996 | 0.7065 | 0.8279 |
| *H. sapiens* | 30 | 4 | Allele detection | 86387 | 84819 | 58 | 8314 | 0.9993 | 0.9122 | 0.9538 |
| *H. sapiens* | 64 | 4 | Allele detection | 89453 | 88296 | 278 | 5248 | 0.9969 | 0.9446 | 0.97 |
| *H. sapiens* | 10 | 6 | Allele detection | 65886 | 65162 | 14 | 28280 | 0.9998 | 0.6997 | 0.8232 |
| *H. sapiens* | 30 | 6 | Allele detection | 84616 | 82588 | 51 | 9550 | 0.9994 | 0.8986 | 0.9463 |
| *H. sapiens* | 50 | 6 | Allele detection | 88118 | 86467 | 252 | 6048 | 0.9971 | 0.9358 | 0.9655 |
| *H. sapiens* | 70 | 6 | Allele detection | 89316 | 87868 | 264 | 4850 | 0.997 | 0.9485 | 0.9721 |
| *H. sapiens* | 97 | 6 | Allele detection | 89797 | 88469 | 293 | 4369 | 0.9967 | 0.9536 | 0.9747 |
| *S. tuberosum* | 60 | 1 | Allele detection | 2247895 | 1957924 | 684776 | 1320370 | 0.7409 | 0.63 | 0.6809 |
| *S. tuberosum* | 30 | 1 | Allele detection | 2168164 | 1885991 | 601867 | 1400101 | 0.7581 | 0.6076 | 0.6746 |
| *S. tuberosum* | 10 | 1 | Allele detection | 1720642 | 1536450 | 407681 | 1847623 | 0.7903 | 0.4822 | 0.599 |
| **FreeBayes (Indel)** | depth | ploidy | Type | True-pos-baseline | True-pos-call | False-pos | False-neg | Precision | Sensitivity | F-measure |
| *H. sapiens* | 10 | 4 | Allele detection | 8668 | 9228 | 5895 | 4085 | 0.6102 | 0.6797 | 0.6431 |
| *H. sapiens* | 30 | 4 | Allele detection | 10466 | 11863 | 10300 | 2287 | 0.5353 | 0.8207 | 0.6479 |
| *H. sapiens* | 64 | 4 | Allele detection | 10945 | 11982 | 9850 | 1808 | 0.5488 | 0.8582 | 0.6695 |
| *H. sapiens* | 10 | 6 | Allele detection | 8284 | 8866 | 6940 | 3925 | 0.5609 | 0.6785 | 0.6141 |
| *H. sapiens* | 30 | 6 | Allele detection | 10043 | 11990 | 16296 | 2166 | 0.4239 | 0.8226 | 0.5595 |
| *H. sapiens* | 50 | 6 | Allele detection | 10623 | 12241 | 18872 | 1586 | 0.3934 | 0.8701 | 0.5419 |
| *H. sapiens* | 70 | 6 | Allele detection | 10910 | 12387 | 19542 | 1299 | 0.388 | 0.8936 | 0.541 |
| *H. sapiens* | 97 | 6 | Allele detection | 11065 | 12460 | 19242 | 1144 | 0.393 | 0.9063 | 0.5483 |
| *S. tuberosum* | 60 | 1 | Allele detection | 305254 | 356249 | 140929 | 111327 | 0.7165 | 0.7328 | 0.7246 |
| *S. tuberosum* | 30 | 1 | Allele detection | 292265 | 346561 | 127485 | 124316 | 0.7311 | 0.7016 | 0.716 |
| *S. tuberosum* | 10 | 1 | Allele detection | 230439 | 258656 | 85874 | 186142 | 0.7508 | 0.5532 | 0.637 |

FreeBayes performance metrics for small-variant genotyping and detection in tetraploid and hexaploid samples under stricter filtering. Each row corresponds to a species, and each column reports evaluation metrics, including precision, recall, and F1 score, for all variants, SNVs, and indels. Results are shown for the relevant ploidy configuration where applicable. Benchmarking was performed using the corresponding truth set for each genome, with chromosome 20 confidence regions used for the human genome. All performance values reflect post-filtered variants within confident regions. See Methods for benchmarking criteria.

**Table S9. Merfin *k*-mer validation in diploids**

|  | Validated | | | Total | | |
| --- | --- | --- | --- | --- | --- | --- |
|  | SNV | Indel | Other | SNV | Indel | Other |
| *F. vesca* | 233,607 | 55,828 | 0 | 233,913 | 56,210 | 0 |
| Validated % | 99.90% | 99.30% |  |  |  |  |
| *Z. mays* | 2,575,758 | 245,962 | 1 | 2,589,324 | 248,328 | 1 |
| Validated % | 99.50% | 99.10% |  |  |  |  |

**Table S10. Mapper replacement with Winnowmap2 and performance summary**

| **GATK (All)** | depth | ploidy | Type | True-pos-baseline | True-pos-call | False-pos | False-neg | Precision | Sensitivity | F-measure |
| --- | --- | --- | --- | --- | --- | --- | --- | --- | --- | --- |
| *H. sapiens* | 32 | 2 | Genotyping | 3830253 | 3860876 | 71930 | 60271 | 0.9817 | 0.9845 | 0.9831 |
| *F. vesca* | 30 | 2 | Genotyping | 259775 | 259800 | 73274 | 29485 | 0.78 | 0.8981 | 0.8349 |
| *S. tuberosum* | 60 | 4 | Genotyping | 2329838 | 2316578 | 1192685 | 1615902 | 0.6601 | 0.5905 | 0.6234 |
| *Z. mays* | 30 | 2 | Genotyping | 1903962 | 1903242 | 5811823 | 928991 | 0.2467 | 0.6721 | 0.3609 |
| **GATK (SNV)** | depth | ploidy | Type | True-pos-baseline | True-pos-call | False-pos | False-neg | Precision | Sensitivity | F-measure |
| *H. sapiens* | 32 | 2 | Genotyping | 3358013 | 3359737 | 14853 | 7102 | 0.9956 | 0.9979 | 0.9967 |
| *F. vesca* | 30 | 2 | Genotyping | 224567 | 222768 | 35886 | 8655 | 0.8613 | 0.9629 | 0.9092 |
| *S. tuberosum* | 60 | 4 | Genotyping | 2141599 | 2105895 | 1032175 | 1393751 | 0.6711 | 0.6058 | 0.6368 |
| *Z. mays* | 30 | 2 | Genotyping | 1758686 | 1750135 | 5265159 | 826376 | 0.2495 | 0.6803 | 0.3651 |
| **GATK (Indel)** | depth | ploidy | Type | True-pos-baseline | True-pos-call | False-pos | False-neg | Precision | Sensitivity | F-measure |
| *H. sapiens* | 32 | 2 | Genotyping | 472240 | 501139 | 57077 | 53169 | 0.8978 | 0.8988 | 0.8983 |
| *F. vesca* | 30 | 2 | Genotyping | 35208 | 37032 | 37388 | 20830 | 0.4976 | 0.6283 | 0.5554 |
| *S. tuberosum* | 60 | 4 | Genotyping | 188239 | 210683 | 160510 | 222151 | 0.5676 | 0.4587 | 0.5074 |
| *Z. mays* | 30 | 2 | Genotyping | 145276 | 153107 | 546664 | 102615 | 0.2188 | 0.586 | 0.3186 |
| **GATK (All)** | depth | ploidy | Type | True-pos-baseline | True-pos-call | False-pos | False-neg | Precision | Sensitivity | F-measure |
| *H. sapiens* | 32 | 2 | Allele detection | 3870534 | 3870498 | 62308 | 19990 | 0.9842 | 0.9949 | 0.9895 |
| *F. vesca* | 30 | 2 | Allele detection | 279140 | 279074 | 54013 | 10120 | 0.8378 | 0.965 | 0.8969 |
| *S. tuberosum* | 60 | 4 | Allele detection | 3032004 | 3007089 | 525233 | 952842 | 0.8513 | 0.7609 | 0.8036 |
| *Z. mays* | 30 | 2 | Allele detection | 2668989 | 2668096 | 5047255 | 163969 | 0.3458 | 0.9421 | 0.5059 |
| **GATK (SNV)** | depth | ploidy | Type | True-pos-baseline | True-pos-call | False-pos | False-neg | Precision | Sensitivity | F-measure |
| *H. sapiens* | 32 | 2 | Allele detection | 3361498 | 3361707 | 12883 | 3617 | 0.9962 | 0.9989 | 0.9976 |
| *F. vesca* | 30 | 2 | Allele detection | 229228 | 226787 | 31877 | 3994 | 0.8768 | 0.9829 | 0.9268 |
| *S. tuberosum* | 60 | 4 | Allele detection | 2773079 | 2709973 | 446223 | 795186 | 0.8586 | 0.7772 | 0.8159 |
| *Z. mays* | 30 | 2 | Allele detection | 2444266 | 2431532 | 4583991 | 140800 | 0.3466 | 0.9455 | 0.5072 |
| **GATK (Indel)** | depth | ploidy | Type | True-pos-baseline | True-pos-call | False-pos | False-neg | Precision | Sensitivity | F-measure |
| *H. sapiens* | 32 | 2 | Allele detection | 509036 | 508791 | 49425 | 16373 | 0.9115 | 0.9688 | 0.9393 |
| *F. vesca* | 30 | 2 | Allele detection | 49912 | 52287 | 22136 | 6126 | 0.7026 | 0.8907 | 0.7855 |
| *S. tuberosum* | 60 | 4 | Allele detection | 258925 | 297116 | 79010 | 157656 | 0.7899 | 0.6215 | 0.6957 |
| *Z. mays* | 30 | 2 | Allele detection | 224723 | 236564 | 463264 | 23169 | 0.338 | 0.9065 | 0.4924 |
| **FreeBayes** |  |  |  |  |  |  |  |  |  |  |
| **FreeBayes (All)** | depth | ploidy | Type | True-pos-baseline | True-pos-call | False-pos | False-neg | Precision | Sensitivity | F-measure |
| *H. sapiens* | 32 | 2 | Genotyping | 3772639 | 3685481 | 307859 | 117885 | 0.9229 | 0.9697 | 0.9457 |
| *F. vesca* | 30 | 2 | Genotyping | 252353 | 239792 | 107807 | 38771 | 0.6899 | 0.8668 | 0.7683 |
| *S. tuberosum* | 60 | 4 | Genotyping | 2723293 | 2208475 | 2455602 | 1228290 | 0.4735 | 0.6892 | 0.5613 |
| *Z. mays* | 30 | 2 | Genotyping | 1562624 | 1462411 | 11334118 | 1270334 | 0.1143 | 0.5516 | 0.1893 |
| **FreeBayes (SNV)** | depth | ploidy | Type | True-pos-baseline | True-pos-call | False-pos | False-neg | Precision | Sensitivity | F-measure |
| *H. sapiens* | 32 | 2 | Genotyping | 3327159 | 3073961 | 24376 | 37956 | 0.9921 | 0.9887 | 0.9904 |
| *F. vesca* | 30 | 2 | Genotyping | 219071 | 193329 | 49368 | 13711 | 0.7966 | 0.9411 | 0.8628 |
| *S. tuberosum* | 60 | 4 | Genotyping | 2459710 | 1686059 | 1395681 | 1082711 | 0.5471 | 0.6944 | 0.612 |
| *Z. mays* | 30 | 2 | Genotyping | 1455807 | 1182121 | 6955264 | 1129259 | 0.1453 | 0.5632 | 0.231 |
| **FreeBayes (Indel)** | depth | ploidy | Type | True-pos-baseline | True-pos-call | False-pos | False-neg | Precision | Sensitivity | F-measure |
| *H. sapiens* | 32 | 2 | Genotyping | 445480 | 611520 | 283483 | 79929 | 0.6833 | 0.8479 | 0.7567 |
| *F. vesca* | 30 | 2 | Genotyping | 33282 | 46463 | 58439 | 25060 | 0.4429 | 0.5705 | 0.4987 |
| *S. tuberosum* | 60 | 4 | Genotyping | 263583 | 522416 | 1059921 | 145579 | 0.3302 | 0.6442 | 0.4366 |
| *Z. mays* | 30 | 2 | Genotyping | 106817 | 280290 | 4378854 | 141075 | 0.0602 | 0.4309 | 0.1056 |
| **FreeBayes (All)** | depth | ploidy | Type | True-pos-baseline | True-pos-call | False-pos | False-neg | Precision | Sensitivity | F-measure |
| *H. sapiens* | 32 | 2 | Allele detection | 3825956 | 3740913 | 252398 | 64568 | 0.9368 | 0.9834 | 0.9595 |
| *F. vesca* | 30 | 2 | Allele detection | 272554 | 257473 | 90126 | 18570 | 0.7407 | 0.9362 | 0.8271 |
| *S. tuberosum* | 60 | 4 | Allele detection | 3491323 | 2821412 | 1846734 | 493523 | 0.6044 | 0.8762 | 0.7153 |
| *Z. mays* | 30 | 2 | Allele detection | 2334846 | 2170083 | 10626445 | 498112 | 0.1696 | 0.8242 | 0.2813 |
| **FreeBayes (SNV)** | depth | ploidy | Type | True-pos-baseline | True-pos-call | False-pos | False-neg | Precision | Sensitivity | F-measure |
| *H. sapiens* | 32 | 2 | Allele detection | 3335500 | 3074581 | 23756 | 29615 | 0.9923 | 0.9912 | 0.9918 |
| *F. vesca* | 30 | 2 | Allele detection | 225566 | 195704 | 46993 | 7216 | 0.8064 | 0.969 | 0.8802 |
| *S. tuberosum* | 60 | 4 | Allele detection | 3183110 | 1951349 | 1130777 | 385155 | 0.6331 | 0.8921 | 0.7406 |
| *Z. mays* | 30 | 2 | Allele detection | 2145685 | 1580384 | 6557001 | 439381 | 0.1942 | 0.83 | 0.3148 |
| **FreeBayes (Indel)** | depth | ploidy | Type | True-pos-baseline | True-pos-call | False-pos | False-neg | Precision | Sensitivity | F-measure |
| *H. sapiens* | 32 | 2 | Allele detection | 490456 | 666332 | 228642 | 34953 | 0.7445 | 0.9335 | 0.8284 |
| *F. vesca* | 30 | 2 | Allele detection | 46988 | 61769 | 43133 | 11354 | 0.5888 | 0.8054 | 0.6803 |
| *S. tuberosum* | 60 | 4 | Allele detection | 308213 | 870063 | 715957 | 108368 | 0.5486 | 0.7399 | 0.63 |
| *Z. mays* | 30 | 2 | Allele detection | 189161 | 589699 | 4069444 | 58731 | 0.1266 | 0.7631 | 0.2171 |

Performance metrics for small-variant genotyping and detection on the human and plant genomes after replacing the mapper with Winnowmap2. Benchmarking was performed using the corresponding truth set for each genome. All performance values reflect post-filtered variants within confident regions. See Methods for benchmarking criteria.

**Table S11. GATK vs DeepVariant on *S. tuberosum* (HiFi)**

| **GATK** | depth | Precision | Sensitivity | F-measure |
| --- | --- | --- | --- | --- |
| *S. tuberosum* | 10x | **0.912** | 0.6666 | 0.7702 |
| *S. tuberosum* | 30x | 0.8923 | 0.7834 | 0.8343 |
| *S. tuberosum* | 60x | 0.8902 | **0.7925** | **0.8385** |
| **DeepVariant** | |  |  |  |
| *S. tuberosum* | 10x | 0.9492 | 0.6485 | 0.7706 |
| *S. tuberosum* | 30x | 0.9572 | **0.7167** | **0.8197** |
| *S. tuberosum* | 60x | **0.9582** | 0.6576 | 0.7799 |

Performance of small variant detection by GATK and DeepVariant on the *S. tuberosum* genome using HiFi reads.

**Table S12. False negatives by genotype in *S. tuberosum* (60×)**

| Genotype | #FNs (DeepVariant) | #FNs (GATK) | The total number |
| --- | --- | --- | --- |
| AAAa | 1078242 (59.3%) | 654961 (36.0%) | 1818863 |
| AAaa | 275882 (25.7%) | 197695 (18.5%) | 1071719 |
| Aaaa | 151736 (15.9%) | 82340 (8.6%) | 952523 |
| aaaa | 8579 (1.5%) | 19636 (3.4%) | 579396 |

Number of false negatives in variant detection by GATK and DeepVariant on the *S. tuberosum* genome, stratified by genotype at 60× sequencing depth.

**Table S13. Compute time and memory usage per caller (~10% genome)**

| Caller | Species | Ploidy | Depth | Wall time (s) | CPU time (s) | Max RSS (MB) |
| --- | --- | --- | --- | --- | --- | --- |
| GATK | *Fragaria vesca* | *2* | 10x | 314.32 | 400.29 | 5061.2 |
| GATK | *Fragaria vesca* | *2* | 30x | 547.34 | 644.21 | 4018.3 |
| GATK | *Zea mays* | *2* | 10x | 7145.95 | 8197.62 | 4232.7 |
| GATK | *Zea mays* | *2* | 30x | 13719.83 | 15151.91 | 3875.8 |
| GATK | *Solanum tuberosum* | *4* | 10x | 8295.91 | 8615.93 | 4987.5 |
| GATK | *Solanum tuberosum* | *4* | 30x | 12150.32 | 12584.62 | 4286.9 |
| GATK | *Solanum tuberosum* | *4* | 60x | 15333.96 | 15998.02 | 3408.6 |
| GATK | *Homo sapiens* | *6* | 10x | 10048.31 | 4484.03 | 4256.8 |
| GATK | *Homo sapiens* | *6* | 30x | 21717.4 | 8473.08 | 3369.4 |
| GATK | *Homo sapiens* | *6* | 60x | 31836.92 | 15275.62 | 3195.1 |
| GATK | *Homo sapiens* | *6* | 70x | 37392.96 | 25266.48 | 3267.6 |
| GATK | *Homo sapiens* | *6* | 90x | 58383.12 | 51860.75 | 5643.6 |
| FreeBayes | *Fragaria vesca* | *2* | 10x | 243.49 | 225.32 | 366.4 |
| FreeBayes | *Fragaria vesca* | *2* | 30x | 800.47 | 795.62 | 972.6 |
| FreeBayes | *Zea mays* | *2* | 10x | 18569.91 | 18461.82 | 863.5 |
| FreeBayes | *Zea mays* | *2* | 30x | 58900.31 | 58674.15 | 806 |
| FreeBayes | *Solanum tuberosum* | *4* | 10x | 6022.81 | 5853.31 | 187.9 |
| FreeBayes | *Solanum tuberosum* | *4* | 30x | 17282.8 | 17256.43 | 690.6 |
| FreeBayes | *Solanum tuberosum* | *4* | 60x | 29278.88 | 29254.79 | 1190.5 |
| FreeBayes | *Homo sapiens* | *6* | 10x | 2990.81 | 1923.94 | 688.8 |
| FreeBayes | *Homo sapiens* | *6* | 30x | 8113.17 | 7324.45 | 564.3 |
| FreeBayes | *Homo sapiens* | *6* | 60x | 9371.91 | 8656.49 | 584.8 |
| FreeBayes | *Homo sapiens* | *6* | 70x | 12924.51 | 12565.68 | 490.4 |
| FreeBayes | *Homo sapiens* | *6* | 90x | 13976.63 | 13571.25 | 1433.1 |

Computational time and memory usage for each caller. Benchmark was conducted on ~10% of the genome using a single workstation. Max RSS was measured per process, and the peak value among all concurrent processes was reported to represent each caller's memory demand.

**Table S14. Hexaploid chr20 validation (trio vs unrelated)**

| **GATK (All)** | depth | ploidy | Type | True-pos-baseline | True-pos-call | False-pos | False-neg | Precision | Sensitivity | F-measure |
| --- | --- | --- | --- | --- | --- | --- | --- | --- | --- | --- |
| HG002 + HG003 + HG004 | 10 | 6 | Genotyping | 48194 | 48319 | 41721 | 58051 | 0.5366 | 0.4536 | 0.4916 |
| HG002 + HG003 + HG004 | 30 | 6 | Genotyping | 76557 | 76886 | 26069 | 29536 | 0.7468 | 0.7216 | 0.734 |
| HG002 + HG003 + HG004 | 50 | 6 | Genotyping | 86330 | 86791 | 18105 | 19707 | 0.8274 | 0.8141 | 0.8207 |
| HG002 + HG003 + HG004 | 70 | 6 | Genotyping | 91248 | 91807 | 13964 | 14760 | 0.868 | 0.8608 | 0.8644 |
| HG002 + HG003 + HG004 | 97 | 6 | Genotyping | 95293 | 95942 | 10423 | 10691 | 0.902 | 0.8991 | 0.9006 |
| HG001 + HG003 + HG004 | 10 | 6 | Genotyping | 48991 | 48990 | 44387 | 72161 | 0.5246 | 0.4044 | 0.4567 |
| HG001 + HG003 + HG004 | 30 | 6 | Genotyping | 85833 | 86262 | 29025 | 35024 | 0.7482 | 0.7102 | 0.7287 |
| HG001 + HG003 + HG004 | 50 | 6 | Genotyping | 96574 | 97140 | 20725 | 24234 | 0.8242 | 0.7994 | 0.8116 |
| HG001 + HG003 + HG004 | 70 | 6 | Genotyping | 102150 | 102728 | 15393 | 18664 | 0.8697 | 0.8455 | 0.8574 |
| HG001 + HG003 + HG004 | 93 | 6 | Genotyping | 106493 | 107224 | 12368 | 14252 | 0.8966 | 0.882 | 0.8892 |
| **GATK (SNV)** | depth | ploidy | Type | True-pos-baseline | True-pos-call | False-pos | False-neg | Precision | Sensitivity | F-measure |
| HG002 + HG003 + HG004 | 10 | 6 | Genotyping | 43961 | 43973 | 34994 | 50170 | 0.5569 | 0.467 | 0.508 |
| HG002 + HG003 + HG004 | 30 | 6 | Genotyping | 70256 | 70292 | 20901 | 23857 | 0.7708 | 0.7465 | 0.7585 |
| HG002 + HG003 + HG004 | 50 | 6 | Genotyping | 79245 | 79286 | 13434 | 14865 | 0.8551 | 0.842 | 0.8485 |
| HG002 + HG003 + HG004 | 70 | 6 | Genotyping | 83872 | 83912 | 9392 | 10231 | 0.8993 | 0.8913 | 0.8953 |
| HG002 + HG003 + HG004 | 97 | 6 | Genotyping | 87678 | 87722 | 5870 | 6425 | 0.9373 | 0.9317 | 0.9345 |
| HG001 + HG003 + HG004 | 10 | 6 | Genotyping | 45170 | 45171 | 38474 | 62778 | 0.54 | 0.4184 | 0.4715 |
| HG001 + HG003 + HG004 | 30 | 6 | Genotyping | 79306 | 79356 | 24288 | 28623 | 0.7657 | 0.7348 | 0.7499 |
| HG001 + HG003 + HG004 | 50 | 6 | Genotyping | 89287 | 89341 | 16460 | 18642 | 0.8444 | 0.8273 | 0.8358 |
| HG001 + HG003 + HG004 | 70 | 6 | Genotyping | 94613 | 94613 | 11253 | 13338 | 0.8937 | 0.8764 | 0.885 |
| HG001 + HG003 + HG004 | 93 | 6 | Genotyping | 98618 | 98683 | 8396 | 9286 | 0.9216 | 0.9139 | 0.9178 |
| **GATK (Indel)** | depth | ploidy | Type | True-pos-baseline | True-pos-call | False-pos | False-neg | Precision | Sensitivity | F-measure |
| HG002 + HG003 + HG004 | 10 | 6 | Genotyping | 4233 | 4346 | 6727 | 7881 | 0.3925 | 0.3494 | 0.3697 |
| HG002 + HG003 + HG004 | 30 | 6 | Genotyping | 6301 | 6594 | 5168 | 5679 | 0.5606 | 0.526 | 0.5427 |
| HG002 + HG003 + HG004 | 50 | 6 | Genotyping | 7085 | 7505 | 4671 | 4842 | 0.6164 | 0.594 | 0.605 |
| HG002 + HG003 + HG004 | 70 | 6 | Genotyping | 7376 | 7895 | 4572 | 4529 | 0.6333 | 0.6196 | 0.6263 |
| HG002 + HG003 + HG004 | 97 | 6 | Genotyping | 7615 | 8220 | 4553 | 4266 | 0.6435 | 0.6409 | 0.6422 |
| HG001 + HG003 + HG004 | 10 | 6 | Genotyping | 3821 | 3819 | 5913 | 9383 | 0.3924 | 0.2894 | 0.3331 |
| HG001 + HG003 + HG004 | 30 | 6 | Genotyping | 6527 | 6906 | 4737 | 6401 | 0.5931 | 0.5049 | 0.5455 |
| HG001 + HG003 + HG004 | 50 | 6 | Genotyping | 7287 | 7799 | 4265 | 5592 | 0.6465 | 0.5658 | 0.6035 |
| HG001 + HG003 + HG004 | 70 | 6 | Genotyping | 7537 | 8115 | 4140 | 5326 | 0.6622 | 0.5859 | 0.6217 |
| HG001 + HG003 + HG004 | 93 | 6 | Genotyping | 7875 | 8541 | 3972 | 4966 | 0.6826 | 0.6133 | 0.6461 |
| **GATK (All)** | depth | ploidy | Type | True-pos-baseline | True-pos-call | False-pos | False-neg | Precision | Sensitivity | F-measure |
| HG002 + HG003 + HG004 | 10 | 6 | Allele detection | 87934 | 88013 | 2284 | 18441 | 0.9747 | 0.8266 | 0.8946 |
| HG002 + HG003 + HG004 | 30 | 6 | Allele detection | 101414 | 101748 | 1923 | 4961 | 0.9815 | 0.9534 | 0.9672 |
| HG002 + HG003 + HG004 | 50 | 6 | Allele detection | 103121 | 103631 | 2182 | 3254 | 0.9794 | 0.9694 | 0.9744 |
| HG002 + HG003 + HG004 | 70 | 6 | Allele detection | 103780 | 104412 | 2390 | 2595 | 0.9776 | 0.9756 | 0.9766 |
| HG002 + HG003 + HG004 | 97 | 6 | Allele detection | 104123 | 104876 | 2623 | 2252 | 0.9756 | 0.9788 | 0.9772 |
| HG001 + HG003 + HG004 | 10 | 6 | Allele detection | 91764 | 91764 | 1644 | 29422 | 0.9824 | 0.7572 | 0.8552 |
| HG001 + HG003 + HG004 | 30 | 6 | Allele detection | 113947 | 114357 | 1815 | 7239 | 0.9844 | 0.9403 | 0.9618 |
| HG001 + HG003 + HG004 | 50 | 6 | Allele detection | 116312 | 116944 | 2014 | 4874 | 0.9831 | 0.9598 | 0.9713 |
| HG001 + HG003 + HG004 | 70 | 6 | Allele detection | 116494 | 116493 | 2045 | 4692 | 0.9828 | 0.9613 | 0.9719 |
| HG001 + HG003 + HG004 | 93 | 6 | Allele detection | 117658 | 118520 | 2340 | 3528 | 0.9806 | 0.9709 | 0.9757 |
| **GATK (SNV)** | depth | ploidy | Type | True-pos-baseline | True-pos-call | False-pos | False-neg | Precision | Sensitivity | F-measure |
| HG002 + HG003 + HG004 | 10 | 6 | Allele detection | 78917 | 78936 | 71 | 15249 | 0.9991 | 0.8381 | 0.9115 |
| HG002 + HG003 + HG004 | 30 | 6 | Allele detection | 91133 | 91153 | 100 | 3033 | 0.9989 | 0.9678 | 0.9831 |
| HG002 + HG003 + HG004 | 50 | 6 | Allele detection | 92663 | 92679 | 104 | 1503 | 0.9989 | 0.984 | 0.9914 |
| HG002 + HG003 + HG004 | 70 | 6 | Allele detection | 93263 | 93278 | 97 | 903 | 0.999 | 0.9904 | 0.9947 |
| HG002 + HG003 + HG004 | 97 | 6 | Allele detection | 93548 | 93564 | 98 | 618 | 0.999 | 0.9934 | 0.9962 |
| HG001 + HG003 + HG004 | 10 | 6 | Allele detection | 83627 | 83631 | 39 | 24347 | 0.9995 | 0.7745 | 0.8728 |
| HG001 + HG003 + HG004 | 30 | 6 | Allele detection | 103551 | 103573 | 124 | 4423 | 0.9988 | 0.959 | 0.9785 |
| HG001 + HG003 + HG004 | 50 | 6 | Allele detection | 105699 | 105720 | 131 | 2275 | 0.9988 | 0.9789 | 0.9887 |
| HG001 + HG003 + HG004 | 70 | 6 | Allele detection | 105860 | 105861 | 27 | 2114 | 0.9997 | 0.9804 | 0.99 |
| HG001 + HG003 + HG004 | 93 | 6 | Allele detection | 106995 | 107016 | 140 | 979 | 0.9987 | 0.9909 | 0.9948 |
| **GATK (Indel)** | depth | ploidy | Type | True-pos-baseline | True-pos-call | False-pos | False-neg | Precision | Sensitivity | F-measure |
| HG002 + HG003 + HG004 | 10 | 6 | Allele detection | 9017 | 9077 | 2213 | 3192 | 0.804 | 0.7386 | 0.7699 |
| HG002 + HG003 + HG004 | 30 | 6 | Allele detection | 10281 | 10595 | 1823 | 1928 | 0.8532 | 0.8421 | 0.8476 |
| HG002 + HG003 + HG004 | 50 | 6 | Allele detection | 10458 | 10952 | 2078 | 1751 | 0.8405 | 0.8566 | 0.8485 |
| HG002 + HG003 + HG004 | 70 | 6 | Allele detection | 10517 | 11134 | 2293 | 1692 | 0.8292 | 0.8614 | 0.845 |
| HG002 + HG003 + HG004 | 97 | 6 | Allele detection | 10575 | 11312 | 2525 | 1634 | 0.8175 | 0.8662 | 0.8411 |
| HG001 + HG003 + HG004 | 10 | 6 | Allele detection | 8137 | 8133 | 1605 | 5075 | 0.8352 | 0.6159 | 0.709 |
| HG001 + HG003 + HG004 | 30 | 6 | Allele detection | 10396 | 10784 | 1691 | 2816 | 0.8644 | 0.7869 | 0.8238 |
| HG001 + HG003 + HG004 | 50 | 6 | Allele detection | 10613 | 11224 | 1883 | 2599 | 0.8563 | 0.8033 | 0.829 |
| HG001 + HG003 + HG004 | 70 | 6 | Allele detection | 10634 | 11354 | 2018 | 2578 | 0.8491 | 0.8049 | 0.8264 |
| HG001 + HG003 + HG004 | 93 | 6 | Allele detection | 10663 | 11504 | 2200 | 2549 | 0.8395 | 0.8071 | 0.8229 |

Performance metrics for small-variant genotyping and detection on the synthetic human hexaploid genome and a synthetic hexaploid of HG001 (instead of HG002). Only GATK was evaluated in this comparison. Benchmarking was performed on chromosome 20 using the corresponding truth set. All performance values reflect post-filtered variants within confident regions. See Methods for benchmarking criteria.
